# Highly effective ammonia removal in a series of Brønsted acidic porous polymers: investigation of chemical and structural variations†
†Electronic supplementary information (ESI) available: Experimental details including synthesis and characterization of polymers, NH_3_ adsorption, breakthrough experiments, and *in situ* infrared spectroscopy. See DOI: 10.1039/c6sc05079d


**DOI:** 10.1039/c6sc05079d

**Published:** 2017-04-27

**Authors:** Gokhan Barin, Gregory W. Peterson, Valentina Crocellà, Jun Xu, Kristen A. Colwell, Aditya Nandy, Jeffrey A. Reimer, Silvia Bordiga, Jeffrey R. Long

**Affiliations:** a Department of Chemistry , University of California , Berkeley , California 94720 , USA . Email: jrlong@berkeley.edu; b Edgewood Chemical Biological Center , U.S. Army Research, Development, and Engineering Command , 5183 Blackhawk Road , Aberdeen Proving Ground , Maryland 21010 , USA; c Department of Chemistry , NIS and INSTM Centre of Reference , University of Turin , Via Quarello 15 , I-10135 Torino , Italy; d Department of Chemical and Biomolecular Engineering , University of California , Berkeley , California 94720 , USA; e Materials Sciences Division , Lawrence Berkeley National Laboratory , Berkeley , California 94720 , USA

## Abstract

Although a widely used and important industrial gas, ammonia (NH_3_) is also highly toxic and presents a substantial health and environmental hazard. The development of new materials for the effective capture and removal of ammonia is thus of significant interest. The capture of ammonia at ppm-level concentrations relies on strong interactions between the adsorbent and the gas, as demonstrated in a number of zeolites and metal–organic frameworks with Lewis acidic open metal sites. However, these adsorbents typically exhibit diminished capacity for ammonia in the presence of moisture due to competitive adsorption of water and/or reduced structural stability. In an effort to overcome these challenges, we are investigating the performance of porous polymers functionalized with Brønsted acidic groups, which should possess inherent structural stability and enhanced reactivity towards ammonia in the presence of moisture. Herein, we report the syntheses of six different Brønsted acidic porous polymers exhibiting –NH_3_Cl, –CO_2_H, –SO_3_H, and –PO_3_H_2_ groups and featuring two different network structures with respect to interpenetration. We further report the low- and high-pressure NH_3_ uptake in these materials, as determined under dry and humid conditions using gas adsorption and breakthrough measurements. Under dry conditions, it is possible to achieve NH_3_ capacities as high as 2 mmol g^–1^ at 0.05 mbar (50 ppm) equilibrium pressure, while breakthrough saturation capacities of greater than 7 mmol g^–1^ are attainable under humid conditions. Chemical and structural variations deduced from these measurements also revealed an important interplay between acidic group spatial arrangement and NH_3_ uptake, in particular that interpenetration can promote strong adsorption even for weaker Brønsted acidic functionalities. *In situ* infrared spectroscopy provided further insights into the mechanism of NH_3_ adsorption, revealing a proton transfer between ammonia and acidic sites as well as strong hydrogen bonding interactions in the case of the weaker carboxylic acid-functionalized polymer. These findings highlight that an increase of acidity or porosity does not necessarily correspond directly to increased NH_3_ capacity and advocate for the development of more fine-tuned design principles for efficient NH_3_ capture under a range of concentrations and conditions.

## Abstract 2

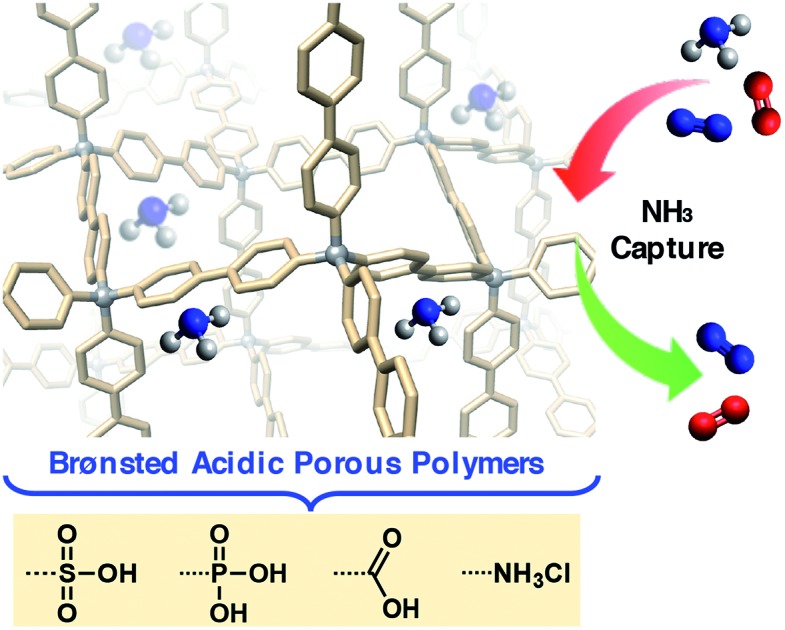
Efficient removal of ammonia from air is demonstrated in a series of Brønsted acidic porous polymers under dry and humid conditions. The impact of acidic group strength and their spatial distribution on the ammonia uptake is investigated systematically.

## Introduction

Solid-state adsorbents such as metal–organic frameworks^1^ and porous polymers^2^ are intriguing classes of materials that have shown significant advancement over the past two decades. Given the flexibility in their design and synthesis, the physical and chemical properties of these materials can be fine-tuned^3^ for a specific application and in particular they have been extensively investigated for applications in gas storage and chemical separations.^4^ In the context of gas capture or separations, it is important to have preferential binding sites with a large enthalpy of adsorption to achieve selective and efficient separation of ppm-level contaminants from the rest of the mixture.^5^

One example where this is of particular importance is in the removal of ammonia (NH_3_) from air.^6^ Ammonia is one of the most highly produced chemicals, and is widely utilized in many essential segments of industry.^7^ On the other hand, ammonia is also highly toxic and poses significant health and environmental concerns.^8^,^9^ For example, CAL-OSHA has limited short-term ammonia exposure to levels as low as 35 ppm while an eight-hour time-weighted average exposure has been limited to 25 ppm.^10^ Effective protection mechanisms against excess ammonia exposure are therefore highly desired in many industrial settings, as well as for military applications. Another potentially interesting application is the removal of residual ammonia in NH_3_-based fuel cells after the gas decomposes to hydrogen.^11^ Indeed, even a small amount of ammonia slip could poison the catalyst and acidic membrane in a fuel cell, and adsorbents that can capture ammonia efficiently at low concentrations could play an important role in the advancement of this technology. In addition to the selective removal of ammonia, porous materials have the potential to exhibit high-capacity storage of ammonia under ambient or high pressures, which would provide a safer alternative to compressed liquid ammonia (10 bar, 25 °C) for transportation and recycling applications.

Ammonia can behave as both a Lewis base and a Brønsted base, and therefore porous materials decorated with Lewis or Brønsted acidic sites are promising targets for capture at particularly low concentrations.^12^ Certain metal–organic frameworks exhibit exposed metal cations, which serve as Lewis acid sites that strongly adsorb ammonia, and these frameworks have been shown to take up high quantities of ammonia at low concentrations under dry conditions.6c,^13^ However, the capacities of these materials are generally significantly diminished in the presence of moisture, a scenario that reflects actual practical operating conditions for ammonia scrubbers.6c,^13^ Such behavior can be attributed to the competition between water and ammonia molecules for Lewis acid sites, as well as to the instability of some of the investigated frameworks under humid conditions. To overcome these problems, a water-stable framework, UiO-66, functionalized with a series of Brønsted acidic groups (–OH, –NH_2_, –CO_2_H, –SO_3_H) was recently investigated for NH_3_ capture.6*g* Although considerable improvement in NH_3_ capacity was achieved using these materials, there was also a significant reduction in porosity upon functionalization with bulkier groups –CO_2_H and –SO_3_H, which hindered the accessibility and complete utilization of the acid sites. More recently, incorporation into a polymer membrane has been demonstrated to impart enhanced stability to the framework HKUST-1 under humid conditions, without diminishing NH_3_ capacity.^14^

Porous polymers have been investigated to a lesser degree in the context of low-pressure NH_3_ capture.^15^ In contrast to metal–organic frameworks, porous polymers inherently exhibit^16^ a high chemical and thermal stability, due to their covalent backbone and accordingly should not suffer from degradation in the presence of ammonia and/or moisture. Furthermore, a diverse toolbox of synthetic organic chemistry allows not only the preparation of polymers with desired porosity (surface area and pore size) but also the incorporation of Brønsted acidic groups in a facile manner. Recently, we demonstrated that a three-dimensional porous polymer functionalized with carboxylic acid groups exhibits a high ammonia uptake of 3.15 mmol g^–1^ at an equilibrium concentration of ∼500 ppm.^17^ The spatial arrangement of multiple carboxylic acids in this material—most likely a result of its multiply-interpenetrated structure—leads to the cooperative reactivity, which in turn creates strong adsorption sites for ammonia at low pressures. Additionally, a sulfonic acid-functionalized porous polymer, prepared from a non-interpenetrated, high-surface area porous aromatic framework (PAF-1), exhibited a lower ammonia capacity of 1.54 mmol g^–1^ at ∼900 ppm, in spite of its stronger Brønsted acidity. This behavior was attributed to the presence of isolated and non-interacting acidic groups due to the non-interpenetrated structure of the material. Such a striking difference between these two polymers prompted us to further investigate the interplay between Brønsted acidity and polymer structure in a systematic manner.

**Fig. 1:**
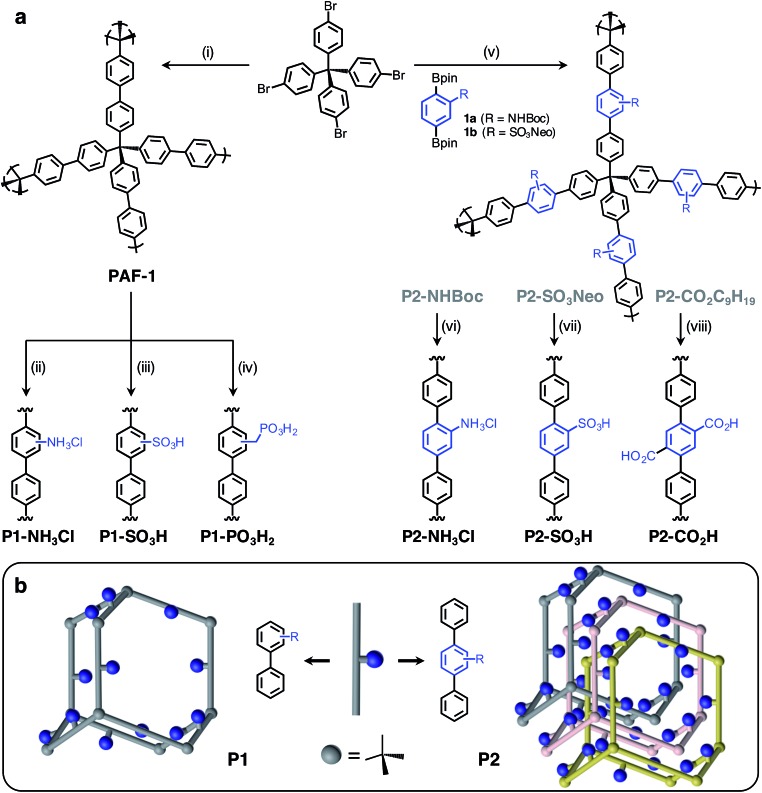
(a) General scheme for the syntheses of P1 and P2 polymers incorporating various Brønsted acid groups. P1 polymers were synthesized using a postsynthetic modification strategy starting from PAF-1, while the P2 polymers were prepared through Suzuki coupling polymerization. Conditions: (i) Ni(cod)_2_, 2,2′-bipyridine, DMF. (ii) Cu(NO_3_)_2_, Ac_2_O, then NaS_2_O_4_; HCl, 1,4-dioxane. (iii) ClSO_3_H, CH_2_Cl_2_. (iv) HCHO, HCl, H_3_PO_4_, AcOH; P(OEt)_3_; Me_3_SiBr, CH_2_Cl_2_, then MeOH. (v) SPhos Pd G2, K_2_CO_3_, H_2_O, THF. (vi) HCl, 1,4-dioxane. (vii) NaN_3_, DMSO, then HCl. (viii) KOH, DMSO. (b) Illustration of non-interpenetrated (P1) and interpenetrated (P2) polymer structures to demonstrate the proximity of Brønsted acidic sites in each structure type.

Herein, we report the synthesis and characterization of six distinct Brønsted acidic porous polymers incorporating –NH_3_Cl, –CO_2_H, –SO_3_H, and –PO_3_H_2_ groups (Fig. 1a) and describe their low- and high-pressure NH_3_ uptake behavior under dry and humid conditions using static gas adsorption and dynamic breakthrough measurements. Through successful incorporation of various acidic groups into non-interpenetrated^18^ (P1) and interpenetrated^19^ (P2) frameworks (Fig. 1b), we were able to investigate the impact of acidic group strength and their spatial arrangement on the overall NH_3_ uptake in a three-dimensionally confined environment. For the best performing materials, we also present the results of *in situ* infrared spectroscopic characterization of the interactions between ammonia and Brønsted acid sites.

## Results and discussion

### Synthesis

The Brønsted acidic porous polymers reported in this work were synthesized following either a postsynthetic functionalization strategy starting from PAF-1 or a *de novo* approach—*i.e.*, first installing Brønsted acidic moieties on one of the monomers and then carrying out a Suzuki coupling polymerization. Preparations for the polymers P1-NH_3_Cl (also known as BPP-2) and P2-CO_2_H (also known as BPP-7) were recently reported,^17^ while the synthesis of P1-SO_3_H (also known as PPN-6-SO_3_H) was achieved following a literature procedure.^20^

Porous polymers P2-NH_3_Cl and P2-SO_3_H were synthesized (Fig. 1a) through the Suzuki coupling route. Precursors **1a** and **1b** were produced starting from 2-amino-1,4-dibromobenzene and 1,4-dibromobenzene, respectively (see ESI†). The *tert*-butyloxycarbonyl (Boc) protection^21^ of the amino group in 2-amino-1,4-dibromobenzene using di-*tert*-butyl dicarbonate was followed by Miyaura borylation to afford **1a**. The sulfonation of 1,4-dibromobenzene using chlorosulfonic acid (ClSO_3_H) provided 2,5-dibromobenzenesulfonyl chloride.^22^ A neopentyl group was chosen as the protecting group in this instance, due to the stability of the resulting sulfonic ester under basic Suzuki–Miyaura coupling conditions, as well as to its ease of removal after polymerization.^23^ Esterification of 2,5-dibromobenzenesulfonyl chloride with neopentyl alcohol and subsequent Miyaura borylation delivered **1b**. Suzuki polymerization of **1a** and **1b** with the tetrahedral precursor tetrakis(4-bromophenyl)methane using Buchwald's precatalyst (SPhos Pd G2) yielded polymers P2-NHBoc and P2-SO_3_Neo, respectively. The utilization of a 4 M HCl solution in 1,4-dioxane enabled the removal of the Boc group and protonation of the amino group simultaneously to yield the final polymer, P2-NH_3_Cl. In the case of P2-SO_3_Neo, hydrolysis of the neopentyl group was carried out using NaN_3_ in DMSO and subsequent acidification with 6 M HCl resulted in the polymer P2-SO_3_H.

Similar to previously reported syntheses of P1-NH_3_Cl and P1-SO_3_H, the polymer P1-PO_3_H_2_ was prepared through postsynthetic modification (Fig. 1a). The parent PAF-1 was synthesized following the original procedure,^24^ while the chloromethylation of PAF-1 to afford the intermediate PAF-1-CH_2_Cl was achieved as reported previously.5*b* The conversion of PAF-1-CH_2_Cl into the ethyl phosphonate polymer P1-PO_3_Et_2_ was accomplished through a Michaelis–Arbuzov reaction in the presence of neat triethyl phosphite, P(OEt)_3_, at elevated temperatures. The mild hydrolysis of the phosphonate ester groups into phosphonic acid moieties was readily accomplished using Me_3_SiBr, affording the polymer P1-PO_3_H_2_ in a quantitative manner.

### Polymer characterization

The formation of porous polymers incorporating protected Brønsted acidic groups and their subsequent hydrolysis was confirmed by Fourier transform infrared (FTIR) spectroscopy (Fig. S7–S9†) and solid-state ^1^H and ^13^C NMR spectroscopy (Fig. S10–S19†). The FTIR spectra of P1-NH_3_Cl, P1-SO_3_H, and P2-CO_2_H were found to be in accordance with previously reported data.^17^,^20^ The formation of P1-PO_3_Et_2_ was confirmed by the appearance of characteristic P=O and P–O–C stretching bands at 1240 and 960–1050 cm^–1^, respectively. The efficiency of this conversion was also reflected in the elemental analysis data (Table S1†). The initial chlorine content of 16.2% in PAF-1-CH_2_Cl was reduced to 0.6% in the subsequent phosphonate ester polymer. Moreover, hydrolysis of P1-PO_3_Et_2_ to P1-PO_3_H_2_ resulted in a shift in the P=O stretching band to 1140 cm^–1^ and the appearance of P–O–H bands in the range of 930–1000 cm^–1^. Complete hydrolysis of ester groups was also evidenced by the absence of P–O–C stretching bands. Similarly, complete removal of the Boc groups in P2-NH_3_Cl was unambiguously demonstrated by the disappearance of a C=O stretching band at 1713 cm^–1^ as well as the emergence of a broad N–H band centered around 3345 cm^–1^. In the case of P2-SO_3_H, absorption bands at 960 cm^–1^ (S–O–C stretching) and at 1178 and 1350 cm^–1^ (S=O stretching) were replaced by bands at 996 and 1020 cm^–1^ (S–OH) and 1150 cm^–1^ (S=O), respectively. Finally, the disappearance of C–H stretching bands in the 2900–3000 cm^–1^ region also indicated the removal of neopentyl groups.

The extent of removal for each protecting group and the structural integrity of P1-PO_3_H_2_, P2-NH_3_Cl, P2-SO_3_H, and P2-CO_2_H were further examined by magic angle spinning (MAS) ^1^H NMR spectroscopy and cross-polarization magic angle spinning (CP/MAS) ^13^C NMR spectroscopy. Acidic protons appear as sharp, narrow peaks, especially in the cases of P1-PO_3_H_2_, P2-SO_3_H, and P2-CO_2_H in the solid-state MAS ^1^H NMR spectra, reflecting the high mobility of acidic protons in these materials (Fig. S12, S16 and S18†). The disappearance of the protons associated with protecting groups further suggested high conversion efficiencies and purities of the final Brønsted acidic porous polymers. The CP/MAS ^13^C NMR spectra also confirmed the hydrolysis of protecting groups by revealing an absence of chemical shifts in the range of 10–40 ppm, for aliphatic carbon atoms on alkyl groups as well as in the 60–80 ppm, region for aliphatic carbon atoms attached to oxygen. Broad chemical shifts in the 110–170 ppm region are ascribed to the presence of aromatic subunits in both protected and final acidic polymers, which also confirm the structural integrity and stability of these polymers under harsh hydrolysis conditions. Scanning electron microscopy images further revealed that the P1 polymers consistently display a sphere-like morphology, while all P2 polymer particles exhibit sheet-like features (Fig. S20 and S21†).

Elemental analyses verified polymer conversion efficiency, with good agreement between experimental and calculated compositions (Tables S1 and S2†), and also enabled quantification of the Brønsted acidic site density within each polymer (Table 1). Accordingly, data for P1-NH_3_Cl and P2-NH_3_Cl revealed a nitrogen content that corresponds to –NH_3_Cl group concentrations of 6.0 and 3.4 mmol g^–1^, respectively. The analysis of sulfur content demonstrated a sulfonic acid loading of 3.7 mmol g^–1^ for P1-SO_3_H and 2.8 mmol g^–1^ for P2-SO_3_H. Phosphorus analysis of P1-PO_3_H_2_ indicated a phosphonic acid concentration of 3.2 mmol g^–1^, which in fact corresponds to 6.4 mmol g^–1^ of available Brønsted acidic sites. Oxygen analysis of P2-CO_2_C_9_H_19_ enabled determination of a carboxylic acid loading of 6.5 mmol g^–1^ in P2-CO_2_H. In the case of the P1 polymers, the number of –NH_3_Cl groups per biphenyl linker (repeating unit) was found to be 1.4 in P1-NH_3_Cl, while the number of –SO_3_H and –PO_3_H_2_ groups was lower in P1-SO_3_H (0.8) and P1-PO_3_H_2_ (0.7), respectively.

**Table 1:** Textural properties including surface areas, pore volumes, and functional group densities of P1 and P2 polymers

	*S*_BET_ ^*a*^ (m^2^ g^–1^)	*S*_micro_ ^*b*^ (m^2^ g^–1^)	*S*_ext_ ^*b*^ (m^2^ g^–1^)	*V*_micro_ ^*b*^ (cm^3^ g^–1^)	*V*_total_ ^*c*^ (cm^3^ g^–1^)	Acid group density^*d*^ (mmol_acid_ g^–1^)
P1-NH_3_Cl	975	833	142	0.33	0.53	6.0
P1-SO_3_H	1220	1035	185	0.41	0.64	3.7
P1-PO_3_H_2_	835	674	161	0.27	0.49	6.4^*e*^
P2-NH_3_Cl	980	835	145	0.33	0.45	3.4
P2-SO_3_H	400	303	97	0.12	0.20	2.8
P2-CO_2_H	715	637	78	0.25	0.30	6.5

^*a*^Brunauer–Emmett–Teller (BET) areas were calculated over the pressure range (*P*/*P*_0_) 0.01–0.06.

^*b*^Micropore/external surface areas and micropore volumes were calculated using the *t*-plot method.

^*c*^Total pore volumes were obtained at *P*/*P*_0_ = 0.95.

^*d*^Density of acidic sites were determined from elemental analysis using the N, S, P, or O content of the corresponding polymer.

^*e*^The value corresponds to twice the number of phosphonic acids to account for its diacidic nature.

### Surface area and pore size distribution

Surface area and porosity analyses were carried out using nitrogen gas adsorption isotherms collected at 77 K. Pore size distributions were calculated from the adsorption branch of isotherms employing a quenched solid-state DFT (QSDFT) model, which takes surface heterogeneity into account and assumes the presence of a mixture of slit, cylindrical, and spherical pores. All polymers display type I reversible isotherms, as typically observed for microporous materials (Fig. S24–S29†) and the overall results from these measurements are summarized in Table 1. The BET surface areas of P1-NH_3_Cl, P1-SO_3_H, and P1-PO_3_H_2_ were found to be 975, 1220, and 835 m^2^ g^–1^, respectively. Since the parent material PAF-1 is considered to be a non-interpenetrated framework owing to its high surface area and expected pore size of ∼11 Å,18*b* postsynthetic functionalization to afford the P1 polymers is expected to preserve this architecture, thus rendering Brønsted acidic sites relatively well-isolated. In spite of the expanded repeating unit length for the P2 polymers (terphenyl instead of biphenyl), the BET surface areas are lower overall than those of the P1 polymers (Table 1). The narrower pore sizes for the P2 polymers, in the range 6–10 Å, also suggests a significant degree of interpenetration and that the Brønsted acidic groups are most likely in close proximity to one another. The relatively high BET surface area for P2-NH_3_Cl (980 m^2^ g^–1^) in this series might be attributed to the presence of the sterically encumbering Boc groups used in the synthesis of the P2-NHBoc precursor, which could reduce the degree of interpenetration.^25^

It should be noted that the total pore volumes for P1 polymers (>0.5 cm^3^ g^–1^) are consistently higher than those for P2 polymers (<0.5 cm^3^ g^–1^), reflecting the influence of interpenetration in the latter frameworks. The microporosity of each sample was also further confirmed by *t*-plot curves (Table 1). All polymers exhibit pore size distributions mainly in the micropore range, while a closer inspection and comparison of pore size distributions for P1 and P2 polymers carrying similar Brønsted acidic groups further reveals some structural differences (Fig. S30–S32†). For instance, although the pore size distributions for P1-NH_3_Cl and P2-NH_3_Cl appear similar, the total volume contribution from pores smaller than 15 Å is slightly larger in P2-NH_3_Cl. This difference becomes more obvious for the polymers comprising –SO_3_H moieties; indeed, while the pore width maxima centered around 6 and 9 Å for P1-SO_3_H are associated with nearly equal pore volume contributions, the major pore volume contribution in P2-SO_3_H arises from the pores centered around ∼6.5 Å. In the case of P1-PO_3_H_2_ and P2-CO_2_H, both materials exhibit a pore-width maximum at ∼6 Å as well as a pore size distribution in the range 7–10 Å, although the overall contribution of 6 Å pores is larger for the interpenetrated polymer P2-CO_2_H, while the 7–10 Å pores contribute more to the pore volume for P1-PO_3_H_2_. Even though these two polymers possess different Brønsted acidic groups, their pore size distributions are in excellent agreement with the anticipated structural features based upon interpenetration.

### NH_3_ adsorption

**Fig. 2:**
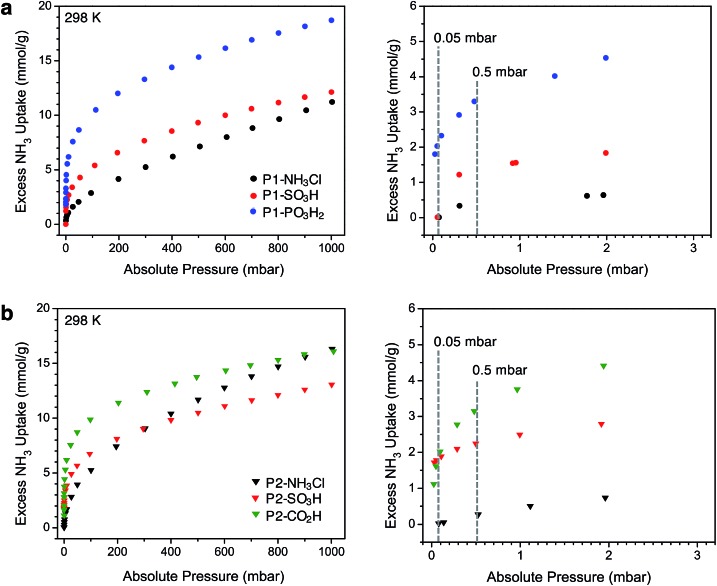
Left panels: NH_3_ adsorption isotherms for (a) P1 (circles) and (b) P2 (triangles) polymers measured at 298 K. Right panels: Low-pressure region for these plots, up to 3 mbar. Dotted gray lines are guides to compare the uptake values at 0.05 mbar (50 ppm) and 0.5 mbar (500 ppm).

After thermally degassing the polymers under vacuum, NH_3_ uptake was investigated at pressures of up to 1 bar at 298 K under dry conditions (Fig. 2). As mentioned earlier, the NH_3_ isotherms for P1-NH_3_Cl, P1-SO_3_H, and P2-CO_2_H were recently reported.^17^ For the sake of a clear discussion and comparison in this section, these isotherms have also been included with those newly collected for P1-PO_3_H_2_, P2-NH_3_Cl, and P2-SO_3_H.

Among the P1 polymers, P1-SO_3_H and P1-PO_3_H_2_ exhibit a significant improvement over P1-NH_3_Cl at low pressures, consistent with the stronger Brønsted acidity of the sulfonic and phosphonic acid groups. For example, at 0.5 mbar (500 ppm), P1-NH_3_Cl displays an excess uptake of 0.37 mmol g^–1^ compared to 1.35 and 3.3 mmol g^–1^, for P1-SO_3_H and P1-PO_3_H_2_, respectively (Fig. 2a). Notably, P1-PO_3_H_2_ shows an uptake of 18.7 mmol g^–1^ at 1 bar, which represents a substantial improvement over the uptakes of 11.2 mmol g^–1^ for P1-NH_3_Cl and 12.1 mmol g^–1^ for P1-SO_3_H at the same pressure. It should be noted that even though phosphonic acid is less acidic than sulfonic acid, P1-PO_3_H_2_ exhibits a steeper uptake in the low-pressure region of the isotherm and higher uptake at low and high pressures under dry conditions. The diacidic nature of this functional group, which increases the total number of acidic sites, and the smaller surface area and pore volume of the associated polymer most likely contribute to the enhanced adsorption under dry conditions. As expected within the series of interpenetrated P2 polymers, P2-SO_3_H and P2-CO_2_H outperform P2-NH_3_Cl in terms of low-pressure NH_3_ uptake (Fig. 2b). The NH_3_ capacity at 0.5 mbar is 2.25 mmol g^–1^ for P2-SO_3_H and 3.15 mmol g^–1^ for P2-CO_2_H, compared to 0.27 mmol g^–1^ for P2-NH_3_Cl. Moreover, despite the weaker acidity of the carboxylic acid groups compared to sulfonic acids, the higher carboxylic acid density (6.5 compared with 2.8 mmol g^–1^ sulfonic acid groups) enables greater NH_3_ uptake at low pressures in P2-CO_2_H—a comparison that also suggests the formation of strong binding sites with multiple weak acidic groups. At 1 bar, P2-NH_3_Cl and P2-CO_2_H display similar NH_3_ uptakes of 16.3 and 16.1 mmol g^–1^, respectively, whereas P2-SO_3_H has a lower uptake of 13.1 mmol g^–1^.

Perhaps more interestingly and relevant to permissible exposure limits, we now compare the uptake properties of these materials at a significantly lower NH_3_ concentration of 50 ppm (0.05 mbar, see right panels in Fig. 2a and b). At this pressure, P1-SO_3_H and P2-SO_3_H exhibit a capacity of 0.01 and 1.79 mmol g^–1^, respectively. Given the lower –SO_3_H loading in P2-SO_3_H (Table 1), it is clear that the spatial distribution and proximity of neighboring acidic groups within the pores is also an important factor that can substantially influence the ammonia affinity of the material. On the other hand, at 50 ppm P1-PO_3_H_2_ and P2-CO_2_H display similarly high NH_3_ uptakes of 2.03 and 1.62 mmol g^–1^, respectively. The improvement in the case of P2-CO_2_H clearly demonstrates that the cooperative action of multiple weaker acidic sites can outperform strong acidic sites that are more isolated. As for P1-PO_3_H_2_, the enhanced NH_3_ uptake relative to P1-SO_3_H is likely a result of: (i) a higher density of acidic sites, (ii) the bulkiness and flexibility of the –CH_2_PO_3_H_2_ groups compared to –SO_3_H groups, and/or (iii) a smaller surface area and pore volume. All of these factors render the acidic sites more proximal in P1-PO_3_H_2_ than in P1-SO_3_H.

Another plausible explanation for the increased NH_3_ uptake in the cases of P1-PO_3_H_2_, P2-SO_3_H, and P2-CO_2_H can be drawn from some recent computational work.12*d* In this study of the interaction of ammonia with isolated Brønsted acidic groups, it was established that protonation of ammonia depends on the dielectric constant of the medium. Given the presence of more isolated sulfonic acid groups in P1-SO_3_H, it is possible that a low local polarity contributes to decreased ammonia affinity at these sites for concentrations as low as 50 ppm. Network interpenetration in the case of P2-SO_3_H and P2-CO_2_H and the reduced pore volume in P1-PO_3_H_2_ could, however, create a local dielectric polarization around each acidic site in the pores and therefore lead to stronger interactions with ammonia and enhanced capacities. Most notably, the performance of these latter materials at 50 ppm of dry ammonia is comparable to that of 5A zeolite (1.86 mmol g^–1^ at 58 ppm) and 13X zeolite (1.74 mmol g^–1^ at 41 ppm) and is significantly higher than sulfonated polymeric resin Amberlyst 15 (0.38 mmol g^–1^ at 71 ppm).^26^

The polymer acidity within the P1 and P2 series, as well as between those with the same functional group (*e.g.*, P1-SO_3_H and P2-SO_3_H), was compared after plotting gravimetric NH_3_ isotherms with respect to the number of acidic functionalities (mmol_NH_3__ mmol_acid_^–1^) determined by elemental analysis. The absolute pressure corresponding to the capture of one equivalent of ammonia per acid site was found to correlate well with the acid strength of functional groups within P1 and P2 (Fig. S33 and S34†). It should be noted that this pressure does not necessarily correspond to complete saturation of acidic sites, since NH_3_ adsorption is typically complex6*d* and capture mechanisms through strong hydrogen bonding and/or van der Waals interactions in small pores cannot be ruled out. Nevertheless, such a comparison is still useful for comparison of the overall binding affinity of the polymers towards ammonia. The abovementioned 1 : 1 interaction is apparent at 12 mbar for P1-PO_3_H_2_, 35 mbar for P1-SO_3_H, and 380 mbar for P1-NH_3_Cl (Fig. S33†), and the lower pressure observed for P1-PO_3_H_2_ compared to P1-SO_3_H can be attributed to reasons discussed above. The 1 : 1 interaction occurs at 2 mbar for P2-SO_3_H, 12 mbar for P2-CO_2_H, and 40 mbar for P2-NH_3_Cl (Fig. S34†), correlating well with the Brønsted acidity of individual sites in these materials. Remarkably, comparison of these pressures between polymers with the same Brønsted acidic functionality reveals the importance of structural features on the overall acidity. For instance, P2-NH_3_Cl exhibits a saturation pressure of 40 mbar compared to a much larger 380 mbar for P1-NH_3_Cl, thus demonstrating that acidic site proximity due to interpenetration can enhance site acidity. Likewise, a similar effect is apparent for P2-SO_3_H and P1-SO_3_H, in which the corresponding pressures are 2 and 35 mbar, respectively.

### Breakthrough measurements

**Fig. 3:**
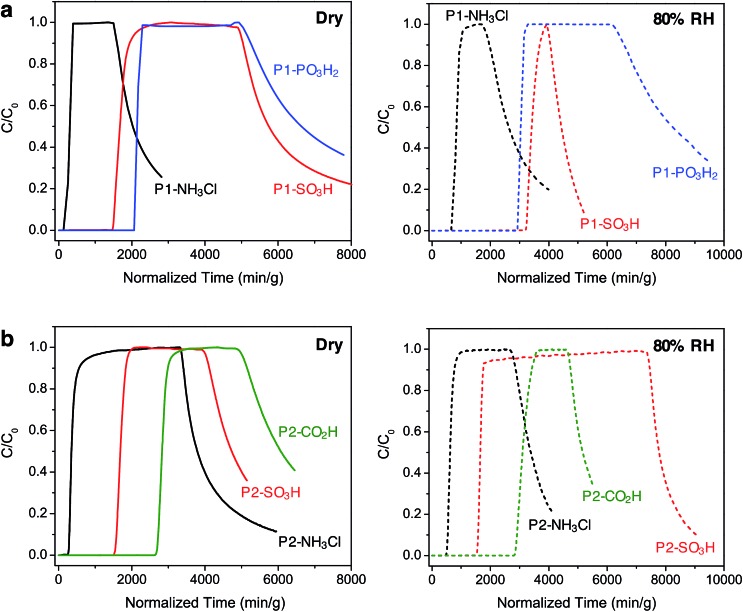
Ammonia breakthrough curves for (a) P1 and (b) P2 polymers under dry (left, solid lines) and humid (right, dashed lines) conditions at 293 K. The challenge concentration (*C*_0_) of ammonia in air was 2000 mg m^–3^ with a flow rate of 20 mL min^–1^. Once saturation was achieved, desorption curves were obtained upon purging the column with air under the corresponding initial dry or humid condition.

In order to gain further understanding of the NH_3_ adsorption in these Brønsted acidic porous polymers and to investigate their removal efficiency under practical conditions, we carried out dynamic microbreakthrough measurements6c,^13^,^27^ under dry and humid conditions at 293 K. The breakthrough curves for the P1 and P2 materials are shown in Fig. 3 and the corresponding capacities upon saturation are summarized in Table 2. The breakthrough curves are plotted on a weighted mass basis to correct for the density of the materials and the partial pressure of ammonia in the feed stream (*C*_0_, 2000 mg m^–3^) was approximately 2.8 mbar.

**Table 2:** Breakthrough capacities of P1 and P2 polymers

	Saturation NH_3_ loadings (mmol g^–1^)	
	Dry (0% RH)	Humid (80% RH)
P1-NH_3_Cl	0.7	2.0
P1-SO_3_H	3.9	8.1
P1-PO_3_H_2_	5.2	7.2
P2-NH_3_Cl	1.0	1.5
P2-SO_3_H	4.0	4.3
P2-CO_2_H	6.7	7.4

Under dry conditions, the trends in breakthrough saturation capacity and uptake are in excellent agreement with those obtained from gas adsorption measurements (Fig. 3a and b, solid lines). For all polymers, the breakthrough curves exhibit a steep slope that suggests that the ammonia capture process is not diffusion controlled. As expected, the breakthrough of ammonia in P1-NH_3_Cl and P2-NH_3_Cl, with the weakest Brønsted acidic functionality, occurs much earlier than for the other polymers carrying sulfonic, phosphonic, or carboxylic acid groups within the same series. Also in agreement with the NH_3_ adsorption isotherms, P1-PO_3_H_2_ and P2-CO_2_H display highest uptake capacities of 5.2 and 6.7 mmol g^–1^, respectively, under dry breakthrough conditions.

We also sought to establish whether the NH_3_ uptake in these materials is reversible, and accordingly monitored the shape of desorption curves obtained upon purging with dry air after ammonia saturation. In the case of the P1 polymers, broad desorption curves suggest a gradual elution of ammonia, while a significant amount of adsorbed ammonia is released from P1-SO_3_H and P1-PO_3_H_2_. In contrast, P2-SO_3_H and P2-CO_2_H retain higher amounts of the gas, as evidenced by their steeper desorption curves. This greater ammonia retention of the interpenetrated P2 polymers can be explained by the presence of a more confined environment around the acidic groups and enhanced interactions with ammonia as a result of the framework interpenetration.

Notably, in the presence of 80% relative humidity (RH), the NH_3_ saturation capacities improve for all polymers when compared with capacities under dry conditions (Fig. 3a and b, dashed lines). The overall capacity increase in the P1 polymers is greater than for the P2 polymers in the presence of water. For example, P1-NH_3_Cl and P1-SO_3_H display saturation capacities of 2.0 and 8.1 mmol g^–1^, respectively, corresponding to more than twice their dry capacities, and P1-PO_3_H_2_ exhibits a humid capacity of 7.2 mmol g^–1^, nearly 1.5 times the uptake under dry conditions. The saturation capacities increase only marginally for P2-NH_3_Cl, P2-SO_3_H, and P2-CO_2_H. Such differences in uptake improvement between the two polymer families can be ascribed to the larger pore volumes of the P1 polymers, which in turn can potentially accommodate more water molecules. Indeed, P1-SO_3_H has the largest total pore volume and exhibits the most significant improvement in NH_3_ uptake. Water adsorption experiments further confirmed a high water uptake of ∼40 mmol g^–1^ for P1-SO_3_H at 80% RH (Fig. S41†). Provided a material is stable under humid conditions, it has previously been found that the presence of water can enhance NH_3_ capacity.^13^,15b,c,^28^ In these cases, dissolution of ammonia in water has been identified as an ammonia retention mechanism.^29^ Furthermore, since the polymers presented here are decorated with polar Brønsted acidic groups, it is possible that: (i) the presence of water facilitates proton transfer between acid sites and ammonia molecules and (ii) dissolution of ammonia is enhanced due to the formation of hydrogen bonding interactions between the polar adsorbent surface and ammonia.^30^ These possible mechanisms highlight the advantage of Brønsted acidic sites over Lewis acidic sites in the context of NH_3_ capture.

We note that while P1-PO_3_H_2_ displays a broad desorption curve under humid conditions, the curve for P1-SO_3_H is rather steep (Fig. 3a, dashed lines). Although ammonia dissolution is a weak retention mechanism and captured ammonia can be released easily upon purging with air,^29^ it appears that the acidity enhancement afforded by water is more pronounced for P1-SO_3_H. On the other hand, ammonia is more strongly held in the P2 polymers, for instance both P2-SO_3_H and P2-CO_2_H exhibit steep desorption curves (Fig. 3b, dashed lines) with a significant ammonia retention. Therefore, depending on whether a high saturation or retention capacity is desired, the polymer structure and chemical features can be adjusted to tune the performance. Based upon humid breakthrough experiments, P1-SO_3_H, P1-PO_3_H_2_, and P2-CO_2_H are identified as the best performing materials with saturation capacities of greater than 7 mmol g^–1^. Importantly, this value surpasses the capacities of a number of other porous polymers (Table S3†). Moreover, the NH_3_ capacities of the Brønsted acidic polymers reported herein are also commensurate with one of the best performing metal–organic frameworks, HKUST-1, which exhibits NH_3_ loadings of 6.6 and 8.9 mmol g^–1^ under dry and humid conditions, respectively.^13^ However, this framework degrades in the presence of moisture and displays diminished NH_3_ uptake after the initial cycle of exposure unless it is embedded^14^ within a polymer membrane. In addition to enhanced uptakes, high structural stabilities of P1-SO_3_H, P1-PO_3_H_2_, and P2-CO_2_H under humid conditions are highly advantageous in terms of recyclability and reusability of these materials in an NH_3_ capture process.^31^

### *In situ* FTIR spectroscopy

We sought to further probe the interaction of ammonia with Brønsted acidic groups utilizing *in situ* infrared spectroscopy. Experiments were carried out on P1-SO_3_H, P1-PO_3_H_2_, and P2-CO_2_H at ambient temperature and at an equilibrium NH_3_ pressure of 3 mbar, corresponding to the partial pressure of ammonia in our breakthrough experiments. Notably, spectral changes were observed at around 1450 cm^–1^ for all three polymers, associated with the formation of the ammonium cation (NH_4_^+^),^32^ as well as changes corresponding to deprotonated species of the Brønsted acidic sites. Thus, at this coverage, a proton transfer mechanism appears to be involved in NH_3_ uptake for these different acidic moieties. Spectral changes were also monitored upon desorption to assess the reversibility of ammonia binding.

We note that the parent material, PAF-1, does not exhibit any substantial spectral changes upon exposure to ammonia (Fig. S37†). The initial spectrum is dominated by the typical features of this aromatic framework, and upon NH_3_ adsorption the spectral changes all corresponded to those associated with the roto-vibrational profile of gaseous ammonia.

**Fig. 4:**
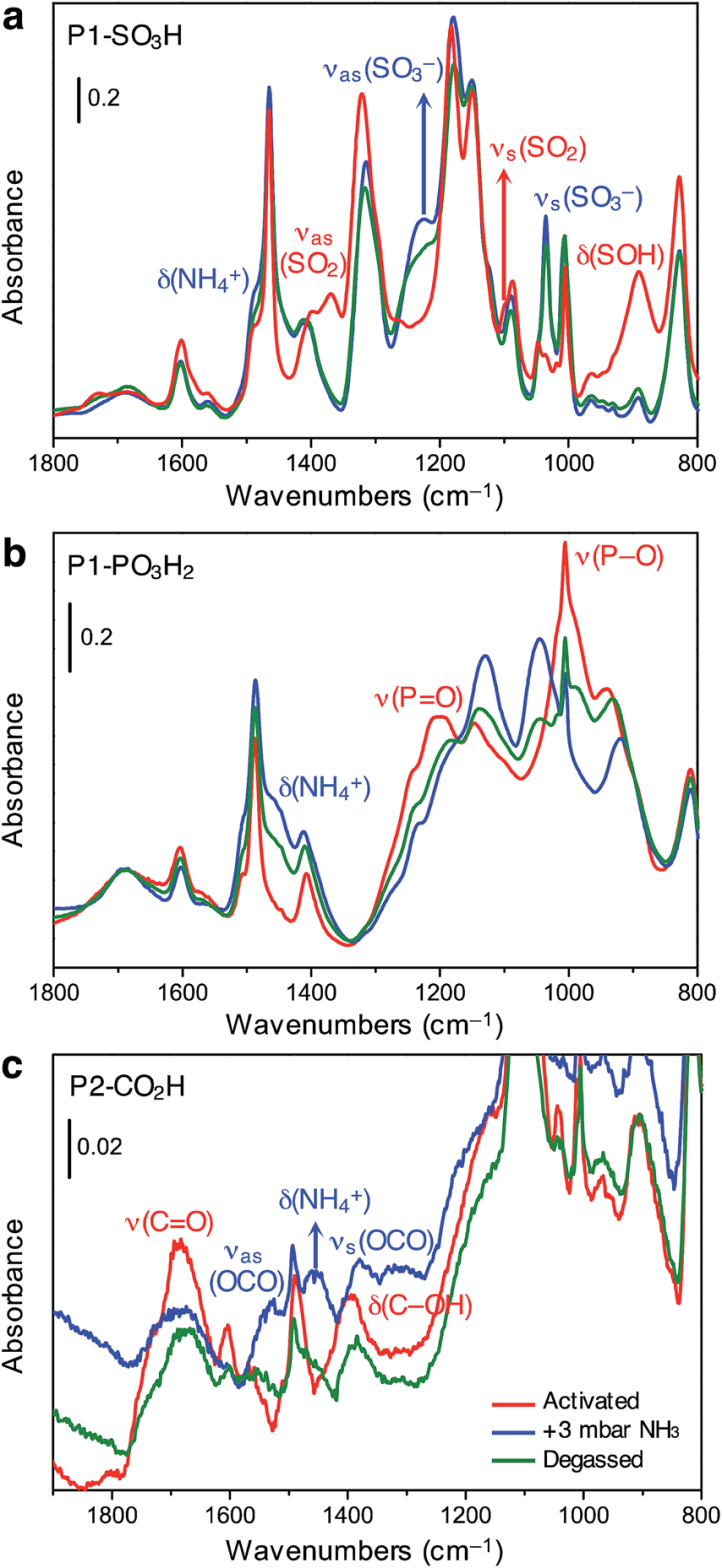
Infrared spectra of (a) P1-SO_3_H, (b) P1-PO_3_H_2_, and (c) P2-CO_2_H in the 1800–800 cm^–1^ spectral range collected at 298 K after its thermal activation under vacuum (red lines), equilibration with ammonia at an equilibrium pressure of 3 mbar (blue lines), and subsequent evacuation under vacuum (green lines).

The spectrum of activated P1-SO_3_H exhibits a series of very intense and sharp absorption bands in the 800–1800 cm^–1^ spectral range, which are ascribed to the vibrational modes of the aromatic structure and sulfonic acid group (Fig. 4a, red curve). In particular, the signals at 1370, 1095, and 895 cm^–1^ are generated by out-of-phase (*ν*_as_) and in-phase (*ν*_s_) stretching vibrations of the SO_2_ group, and the OH bending mode of the S–OH moiety, respectively.^32^ After equilibrating the sample with 3 mbar of ammonia, important spectral modifications become apparent (Fig. 4a, blue curve). A new broad signal emerges in the 1440–1490 cm^–1^ region as a consequence of the formation of ammonium ions and overlaps with one of the peaks resulting from aromatic ring vibrations at 1465 cm^–1^. The absorption bands assigned to SO_2_ and S–OH moieties in the activated material also disappear and two strong signals appear at 1035 and 1225 cm^–1^ due to *ν*_s_ and *ν*_as_ modes of the newly formed sulfonate (–SO_3_^–^) group.^32^ All of these spectral changes indicate that proton transfer to ammonia indeed occurs at 3 mbar. After NH_3_ adsorption, the sample was evacuated for 2 h at beam temperature (residual pressure < 10^–4^ mbar) to evaluate the reversibility of the proton transfer (Fig. 4a, green curve). The aforementioned absorption bands at 1035, 1225, and 1485 cm^–1^ remain almost unchanged upon desorption, demonstrating the high stability of the generated ammonium ion. Additionally, the *ν*_as_(SO_2_) and *ν*_s_(SO_2_) modes of the sulfonic acid moieties could not be restored, even after prolonged outgassing.

Spectra collected for *in situ* ammonia-dosed on P1-PO_3_H_2_ suggested a similar proton transfer between ammonia and phosphonic acid groups within the polymer. The activated spectrum of P1-PO_3_H_2_ exhibits typical features, in particular, strong and broad signals located at around 1000 and 1200 cm^–1^ and corresponding to P–O(H) and P=O stretching vibrations, respectively (Fig. 4b, red curve).^32^ Upon dosing with ammonia, the *δ*(NH_4_^+^) mode of the ammonium ion clearly appears as a broad component at ∼1450 cm^–1^, while the P–O and P=O stretching modes are deeply perturbed, indicating deprotonation of one or both acidic protons (Fig. 4b, blue curve). Interestingly, outgassing following NH_3_ adsorption results in a partial restoration of the spectral features of the activated sample (Fig. 4b, green curve).

The spectrum of activated P2-CO_2_H exhibits two broad signals at 1688 and 1393 cm^–1^, which we assign to *ν*(C=O) stretching and *δ*(C–OH) bending vibrations of the carboxylic acid group, respectively (Fig. 4c, red curve).^32^ Exposure of the sample to 3 mbar of ammonia leads to immediate appearance of the *δ*(NH_4_^+^) mode at ∼1458 cm^–1^ (Fig. 4c, blue curve). Moreover, the *ν*(C=O) and *δ*(C–OH) band intensities decrease, and this change is accompanied by the appearance of two new bands at 1525 and 1378 cm^–1^ that can be ascribed to *ν*_as_(OCO) and *ν*_s_(OCO) modes of the carboxylate (–CO_2_^–^) group, respectively.^32^ Notably, in contrast to what was observed for P1-SO_3_H and P1-PO_3_H_2_, for P2-CO_2_H, the proton transfer to ammonia seems to be quite reversible (Fig. 4c, green curve). Indeed, after outgassing the ammonia-exposed sample, the *δ*(NH_4_^+^) and *ν*(OCO) bands decrease dramatically and the characteristic signals of the carboxylic acid groups are partially restored.

Another unique feature exhibited by P2-CO_2_H upon ammonia exposure is the noticeable change in the scattering profile of the whole spectrum (Fig. S40†). The spectrum displays an evident increase in the absorption profile below 3000 cm^–1^, indicative of very strong hydrogen bonding. In conjunction with the partially reversible appearance of the ammonium and carboxylate signals, this feature is indicative of the presence of a hesitating proton with high mobility—*i.e.*, the proton fluctuates between carboxylic acid and ammonia.^33^ Importantly, this observation sheds further light on the enhanced NH_3_ adsorption capacity of P2-CO_2_H over P1-SO_3_H at low pressures, as discussed earlier. In spite of a weaker acidity, the high density of carboxylic acid groups in an interpenetrated structure creates strong binding sites for ammonia as well as a stabilizing polar environment for ammonium ions.

## Conclusions

The foregoing results demonstrate the efficient removal of ammonia from air in two series of Brønsted acidic porous polymers, one without (P1) and one with (P2) framework interpenetration, wherein the strength of the acidic functional groups was systematically varied. Adsorption isotherms revealed that NH_3_ capacities of the P1-PO_3_H_2_, P2-SO_3_H, and P2-CO_2_H polymers are competitive with those of traditional adsorbents, such as zeolites, and are significantly better than acidic polymer resins, particularly in the low pressure region. Furthermore, dynamic breakthrough experiments performed under humid conditions, which are more relevant to air filtration applications, also revealed that P1-SO_3_H, P1-PO_3_H_2_, and P2-CO_2_H outperform most other metal–organic frameworks and porous polymers reported to date for NH_3_ capture (Tables S3 and S4†). These findings emphasize the advantage of Brønsted acidic over Lewis acidic sites, specifically due to the competition between ammonia and water adsorption that has been observed in zeolites and metal–organic frameworks with open metal coordination sites. Furthermore, study of the mechanism of NH_3_ adsorption in P1-SO_3_H, P1-PO_3_H_2_, and P2-CO_2_H *via in situ* infrared spectroscopy revealed a proton transfer reaction as well as strong hydrogen bonding interactions in the case of P2-CO_2_H. Most strikingly, the proximity of multiple weaker acidic groups in the interpenetrated polymer P2-CO_2_H and their cooperative reactivity amplifies the strength of interaction with ammonia molecules. The NH_3_ uptake in P2-CO_2_H is thus larger than for P1-SO_3_H, which exhibits stronger-binding but more isolated acid groups. Taking both the low- and high-pressure uptake behavior of these polymers into account, it can be concluded that both the Brønsted acidity strength and pore environments govern the amount of ammonia adsorbed, while humidity and corresponding water co-adsorption also plays an important role in the NH_3_ uptake mechanism. More broadly, the high stability of porous polymers and the level of synthetic control demonstrated here suggests that these materials may present excellent platforms for a range of desired capture and storage applications, for ammonia as well as other toxic gases.

## Supplementary Material

Supplementary information
