# Supplementary material for: Highly effective ammonia removal in a series of Brønsted acidic porous polymers: investigation of chemical and structural variations
Source: Chem Sci. 2017 Apr 27;8(6):4399–409. doi: 10.1039/c6sc05079d (PMC6100238; doi:10.1039/c6sc05079d)
Supplement: Supplementary file 1 [file SC-008-C6SC05079D-s001.pdf]

Electronic Supplementary Material (ESI) for Chemical Science.
This journal is © The Royal Society of Chemistry 2017

Highly Effective Ammonia Removal in a Series of Brønsted Acidic Porous Polymers: Investigation of Chemical and Structural Variations

Gokhan Barin,[a] Gregory W. Peterson,[b] Valentina Crocellà,[c] Jun Xu,[d] Kristen A. Colwell,[d] Aditya Nandy,[d] Jeffrey A. Reimer,[de] Silvia Bordiga,[c] Jeffrey R. Long*[ade]

[a]*Department of Chemistry and* [d]*Department of Chemical and Biomolecular Engineering, University of California, Berkeley, California 94720, USA*

[b]*Edgewood Chemical Biological Center, U.S. Army Research, Development, and Engineering Command, 5183 Blackhawk Road, Aberdeen Proving Ground, Maryland 21010, USA*

[c]*Department of Chemistry, NIS and INSTM Centre of Reference, University of Turin, Via Quarello 15, I-10135 Torino, Italy*

[e]*Materials Sciences Division, Lawrence Berkeley National Laboratory, Berkeley, California 94720, USA*

*Email: *jrlong@berkeley.edu*

SUPPORTING INFORMATION

Table of Contents

1. General Methods………………………………………………………………… **S2**
2. Synthesis………………………………………………………………………… **S3**
3. Structural Characterization of Polymers……………………………………….. **S11**
  3.1. Elemental Analysis……………………………………………………... **S11**
  3.2. FTIR Spectroscopy……………………………………………………... **S12**
  3.3. Solid-State NMR Spectroscopy……………………………………....... **S13**
  3.4. Scanning Electron Microscopy………………………………………… **S19**
  3.5. Thermogravimetric Analysis…………………………………………… **S20**
  3.6. Surface Area and Pore Size Distributions……………………………. **S21**
4. NH_3 Adsorption…………………………………………………………….. **S25**
5. Breakthrough Experiments………………………………………………....... **S27**
6. *In situ* Infrared Spectroscopy……………………………………………...... **S28**
7. Water Isotherm for P1-SO_3H…………………………………………......... **S30**
8. List of Porous Materials and Their NH_3 Uptake Capacities……………… **S31**
9. References……………………………………………………………………. **S34**

1. General Methods

Starting materials and reagents were purchased from Sigma-Aldrich and used as received without further purification. Tetrakis(4-bromophenyl)methane,[1] **S1**,[2] **S2**,[3] PAF-1,[4] PAF-1-CH_2Cl,[5] P1-NH_3Cl,[6] P1-SO_3H,[7] P2-$CO_2C_9H_{19}$,[6] and P2-CO_2H[6] were prepared following the procedures reported in the literature. All reactions were performed under a nitrogen or argon atmosphere and in dry solvents, unless otherwise stated. Analytical thin-layer chromatography (TLC) was performed on aluminum sheets, precoated with silica gel 60-F_{254} (Merck 5554). Flash column chromatography was carried out using silica gel 60 (Silicycle) as the stationary phase. Deuterated solvents were purchased from Cambridge Isotope Laboratories (Andover, MA) and used without further purification. ^1H and ^{13}C NMR spectra were recorded on a Bruker AV-400 and Bruker AMX 400 spectrometers (400.132 MHz for ^1H and 100.623 MHz for ^{13}C) at ambient temperature.

^1H NMR data are reported as follows: chemical shift (multiplicity (br s = broad singlet, s = singlet, d = doublet, dd = doublet of doublets), coupling constants, and integration). Chemical shifts are reported in ppm relative to the signals corresponding to the residual non-deuterated solvents. Electrospray Ionization (ESI) mass spectra were obtained from QB3/Chemistry Mass Spectrometry Facility at the University of California, Berkeley.

Thermal gravimetric analysis (TGA) data was collected at ramp rates of 5 ºC/min under flowing nitrogen using a TA Instruments TGA Q5000. Infrared spectra were obtained on a Perkin-Elmer Spectrum 100 Optica FTIR spectrometer furnished with an attenuated total reflectance accessory. Carbon, hydrogen, nitrogen, and sulfur elemental analyses were obtained from the Microanalytical Facility at the University of California, Berkeley. Elemental analyses for chlorine, phosphorus, and oxygen were performed at Galbraith Laboratories.

Scanning electron microscopy (SEM) samples of polymers were prepared by dispersing fine powders into methanol and drop casting onto a silicon chip. To dissipate charge, the samples were sputter coated with approximately 3 nm of Au (Denton Vacuum). Polymers were imaged at 5 keV and 12 μA by field emission SEM (JEOL FSM6430).

Solid-state ^1H–^{13}C cross-polarization (CP) spectra were collected on a 7.05 Tesla magnet at ^{13}C frequency of 75.5 MHz under 10 kHz magic-angle spinning (MAS) condition. A Chemagnetics 4 mm H/X probe and a Tecmag Discovery spectrometer were used. The Hartmann-Hahn condition for CP experiments was obtained on solid adamantane, which is also a secondary reference of ^{13}C chemical shift (the methylene signal of adamantane was set to 38.48 ppm relative to TMS). Two pulse phase modulation (TPPM) proton decoupling scheme was used. The TPPM angle was 15 degrees and the decoupling field strength was ~60 kHz. A contact time of 10 ms and a pulse delay of 4 s were used in CP experiments. Solid-state ^1H NMR spectra were also collected using the same instrument under 13.5 kHz MAS condition. The ^1H chemical shift was calibrated on adamantine (1.74 ppm relative to TMS). A 90-degree pulse of 3.3 μs and a pulse delay of 4 s were used in ^1H MAS experiments. Experimental ^1H MAS NMR spectra were deconvoluted to show individual peaks.

Gas adsorption isotherms were measured using a Micromeritics ASAP 2020/2420 or 3Flex instruments. Samples were transferred to a pre-weighed glass analysis tube, which was capped with a Transeal, and were evacuated on the degas ports until the outgas rate was less than 3 μbar/min. Ultrahigh-purity grade (99.999%) nitrogen and anhydrous ammonia (99.999%) was used for gas adsorption measurements. Ammonia and water isotherms were obtained at 25 ºC and 20 ºC, respectively, using a water circulator. Nitrogen isotherms were obtained using a 77 K liquid-N_2 bath and used to determine the surface areas and pore volumes using the Micromeritics software, assuming a value of 16.2 $Å^2$ for the molecular cross-sectional area of N_2. Pore-size distributions were calculated using the density functional theory method with a QSDFT adsorption branch model of N_2 at 77 K adsorbed in carbon with slit/cylindrical/spherical pores, as implemented in the Quantachrome *VersaWin* software. The activation temperatures for porous polymers, except those mentioned in the following Synthesis section, were: 150 ºC for PAF-1, 120 ºC for PAF-1-CH_2Cl, 110 ºC for P1-NH_3Cl, 120 ºC for P1-SO_3H, and 110 ºC for P2-CO_2H.

2. Synthesis

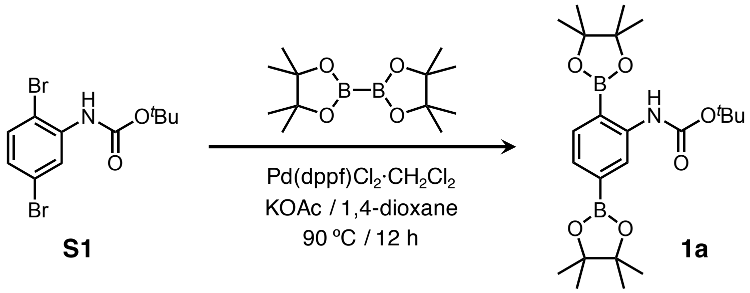

Scheme S1 Synthesis of compound **1a**

Synthesis of 1a: A 100-mL 3-neck round bottomed flask was charged with compound **S1** (2 g, 5.7 mmol), bis(pinacolato)diboron (4.4 g, 17.3 mmol), KOAc (3.4 g, 34.6 mmol), and Pd(dppf)Cl_2·CH_2Cl_2 (450 mg, 0.55 mmol) and then equipped with a reflux condenser and rubber stoppers. The solid mixture was subjected to three cycles of brief vacuum/argon (degas/backfill) with no stirring. Anhydrous 1,4-dioxane (50 mL) was quickly transferred into the flask using a syringe under argon atmosphere and the suspension was stirred for 12 h at 90 ºC. After confirming the completion of reaction by TLC (EtOAc/Hexanes 5:1), the reaction mixture was cooled down to room temperature and concentrated *in vacuo*. The residual solid was redissolved in $CHCl_3$ (50 mL), washed with H_2O (50 mL), dried over $MgSO_4$, and suspended over activated carbon for 1 h, after which was filtered through a pad of Celite. The solvent was removed under reduced pressure and remaining solid was briefly washed with EtOAc and MeOH to deliver compound **1a** as a pale yellow powder (1.5 g, 59%). ^{1}H NMR ($CDCl_3$, 400 MHz, 298 K): δ = 8.60 (br s, 1H), 8.51 (s, 1H), 7.70 (d, *J* = 7.4 Hz, 1H), 7.40 70 (d, *J* = 7.4 Hz, 1H), 1.53 (s, 9H), 1.36 (s, 12H), 1.33 (s, 12H); ^{13}C NMR ($CDCl_3$, 101 MHz, 298 K): δ = 153.07, 144.53, 135.33, 127.68, 123.58, 84.28, 83.85, 79.61, 28.41, 24.89; HRMS (ESI) *m/z* calcd 468.2699 for $C_{23}H_{37}O_6NB_2Na$ $[M+Na]^+$, found 468.2701.

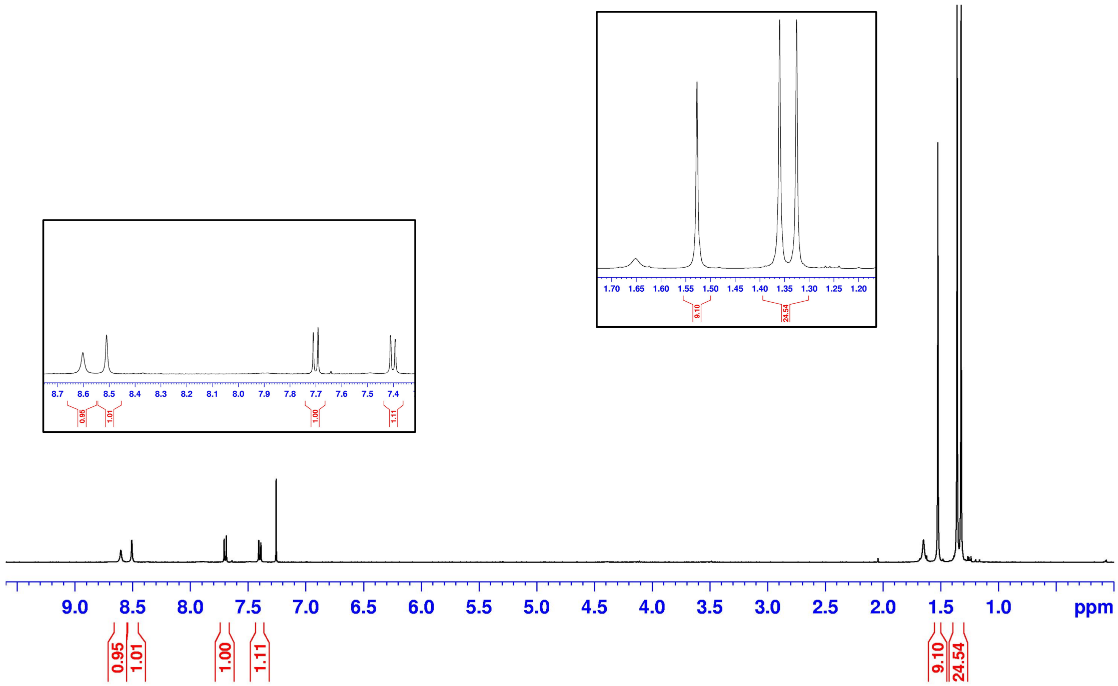

Fig. S1 ^{1}H NMR spectrum of **1a** recorded in $CDCl_3$.

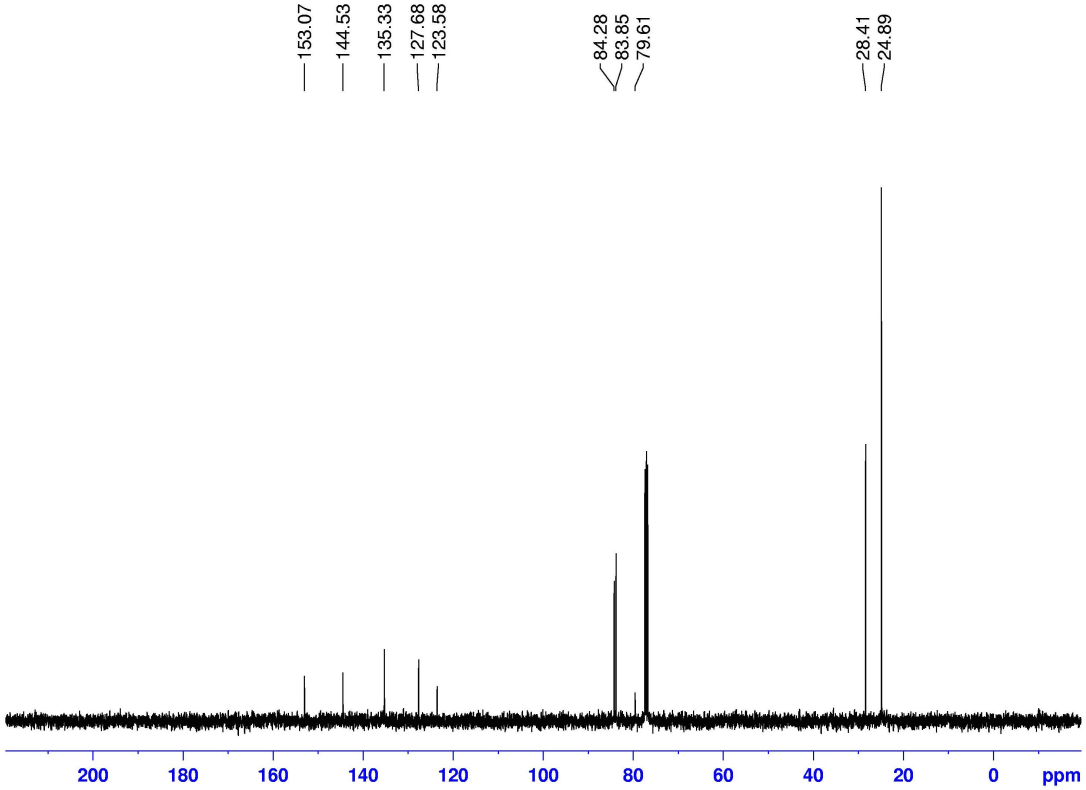

Fig. S2 ^{13}C NMR spectrum of **1a** recorded in $CDCl_3$.

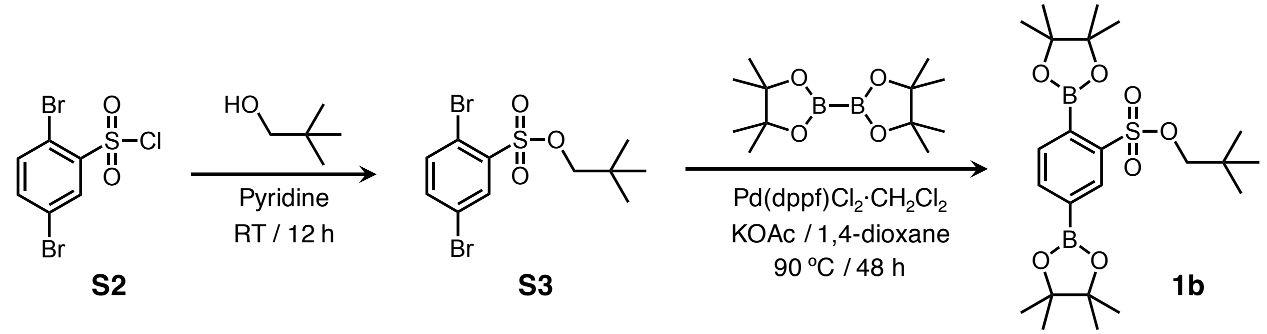

Scheme S2 Synthesis of compound **1b**

Synthesis of S3: The procedure was adopted from a literature report.[8] Compound **S2** (5 g, 15.0 mmol) and neopentyl alcohol (1.58 g, 17.9 mmol) were dissolved in CH_2Cl_2 (25 mL) and cooled to 0 ºC in an ice-water bath. Then, pyridine (2.5 mL, 30.0 mmol) was added dropwise over a period of 30 min. The reaction mixture was allowed to stir at room temperature for 12 h and diluted with Et_2O. The organic layer was washed with 0.1% HCl followed by brine and then dried over $MgSO_4$. After removing the solvent *in vacuo*, recrystallization of the crude product in EtOH provided **S3** as a colorless crystalline solid (3.7 g, 64%). ^{1}H NMR ($CDCl_3$, 400 MHz, 298 K): δ = 8.24 (d, *J* = 2.3 Hz, 1H), 7.65 (d, *J* = 8.4 Hz, 1H), 7.59 (dd, *J* = 8.4, 2.3 Hz, 1H), 3.77 (s, 2H), 0.98 (s, 9H); ^{13}C NMR ($CDCl_3$, 101 MHz, 298 K): δ = 137.51, 136.96, 134.64, 121.42, 119.48, 81.01, 31.80, 26.13; HRMS (ESI) *m/z* calcd 312.8175 for $C_6H_3O_3Br_2S$ $[M\text{–}C_5H_{11}]^-$, found 312.8176.

Synthesis of 1b: A 100-mL 3-neck round bottomed flask were charged with compound **S3** (1.5 g, 3.9 mmol), bis(pinacolato)diboron (2.96 g, 11.7 mmol), KOAc (2.25 g, 22.9 mmol), and $Pd(dppf)Cl_2{\cdot}CH_2Cl_2$ (350 mg, 0.43 mmol) and then equipped with a reflux condenser and rubber stoppers. The solid mixture was subjected to three cycles of brief vacuum/argon (degas/backfill) with no stirring. Anhydrous 1,4-dioxane (40 mL) was quickly transferred into the flask using a syringe under argon atmosphere and the suspension was stirred for 48 h at 90 ºC. After confirming the completion of reaction by TLC, the reaction mixture was cooled down to room temperature and concentrated *in vacuo*. The residual solid was redissolved in $CHCl_3$ (50 mL), washed with H_2O (50 mL), dried over $MgSO_4$, and suspended over activated carbon for 1 h, after which was filtered through a pad of Celite. The solvent was removed under reduced pressure, followed by the addition of MeOH and the flask was placed in a −30 ºC freezer to induce precipitation. The product **1b** was collected by filtration as a yellow powder (0.8 g, 43%). ^{1}H NMR ($CDCl_3$, 400 MHz, 298 K): δ = 8.35 (s, 1H), 7.97 (dd, *J* = 7.4, 0.8 Hz, 1H), 7.57 (d, *J* = 7.4 Hz, 1H), 3.76 (s, 2H), 1.40 (s, 12H), 1.36 (s, 12H), 0.92 (s, 9H); ^{13}C NMR ($CDCl_3$, 101 MHz, 298 K): δ = 138.40, 138.34, 134.16, 132.57, 84.74, 84.43, 79.56, 31.70, 26.08, 24.90; HRMS (ESI) *m/z* calcd 503.2417 for $C_{23}H_{38}O_7B_2SNa$ $[M+Na]^+$, found 503.2419.

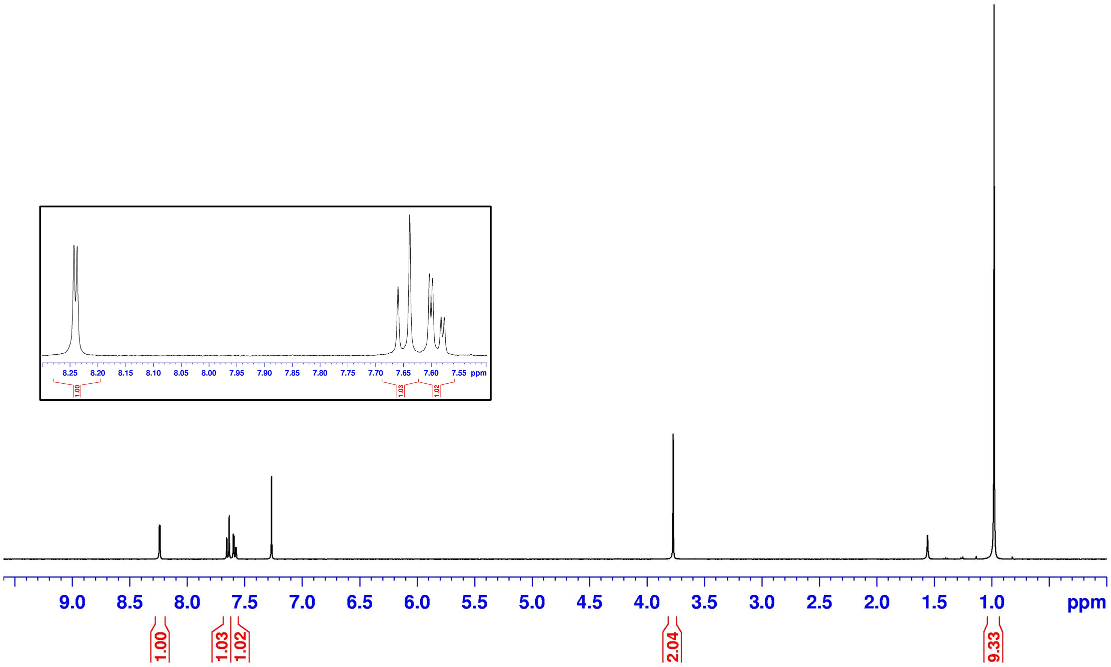

Fig. S3 ^{1}H NMR spectrum of **S3** recorded in $CDCl_3$.

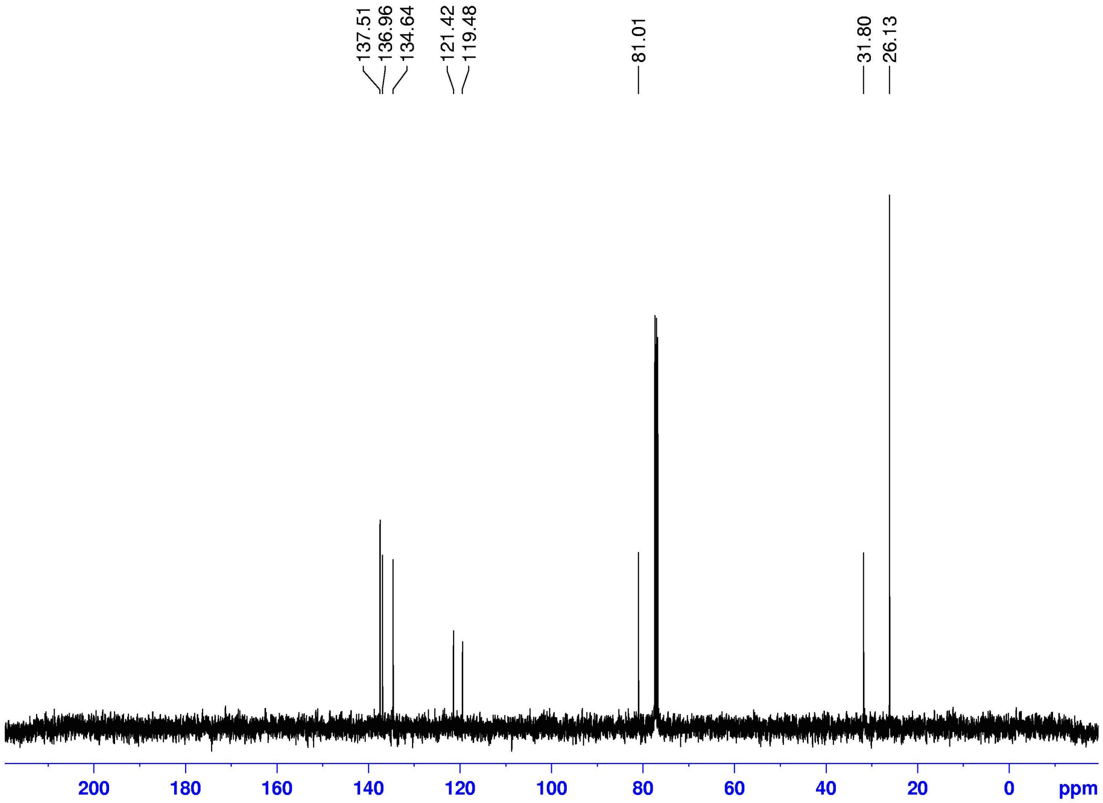

Fig. S4 ^{13}C NMR spectrum of **S3** recorded in $CDCl_3$.

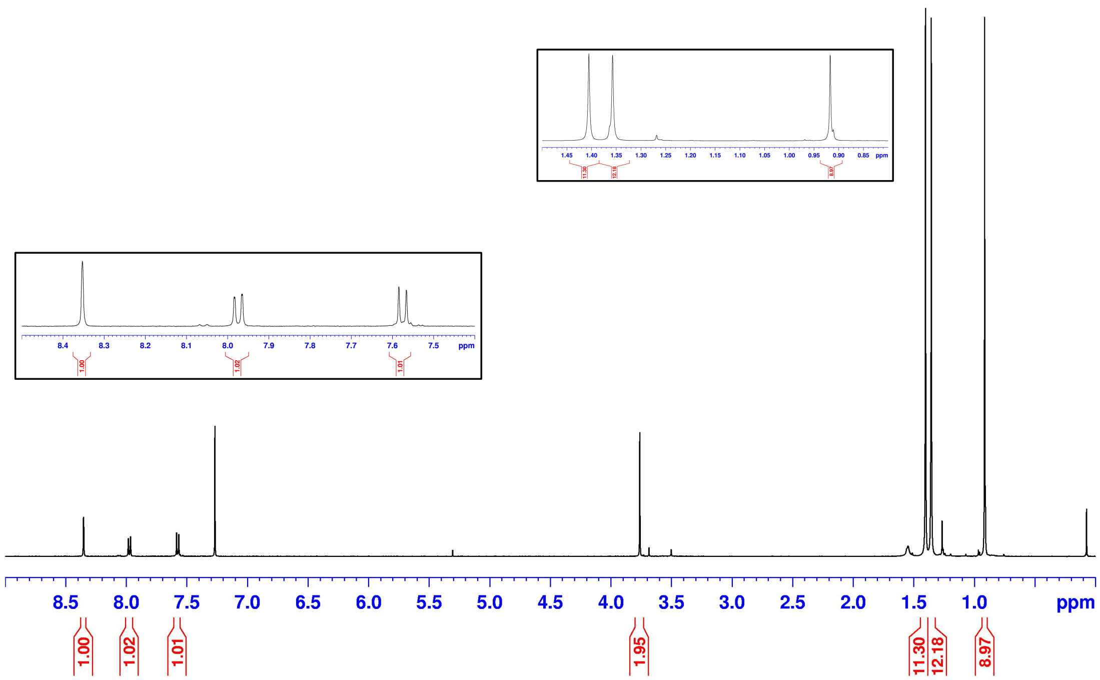

Fig. S5 ^{1}H NMR spectrum of **1b** recorded in $CDCl_3$.

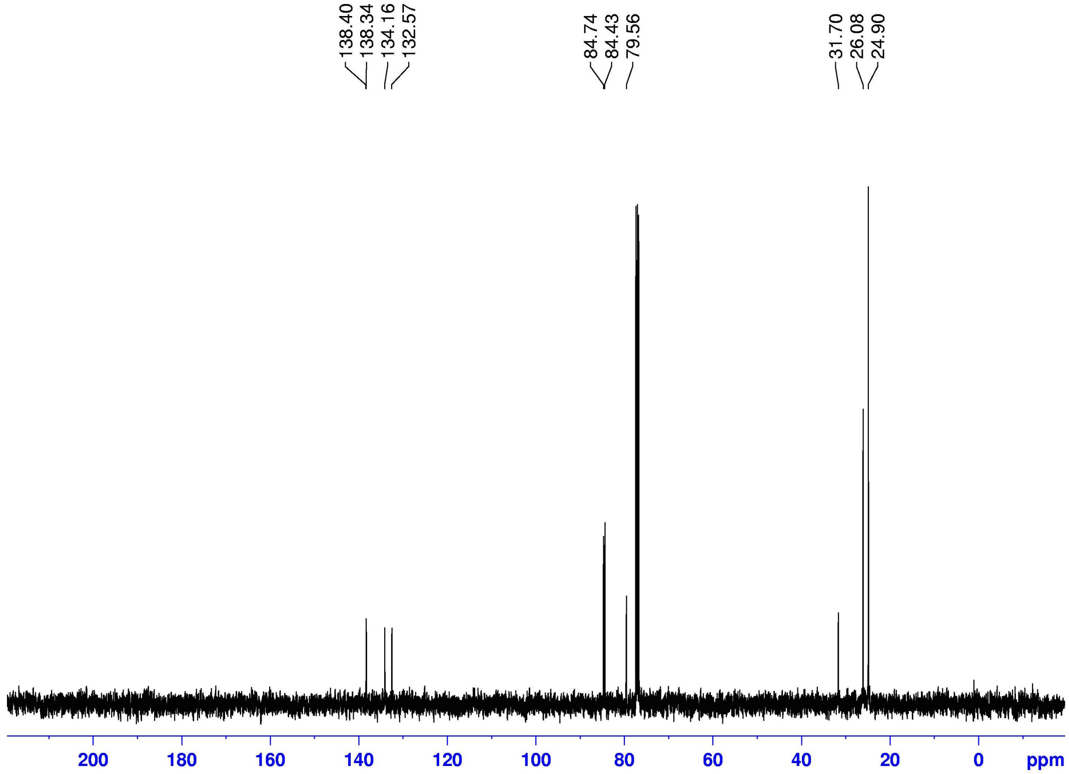

Fig. S6 ^{13}C NMR spectrum of **1b** recorded in $CDCl_3$.

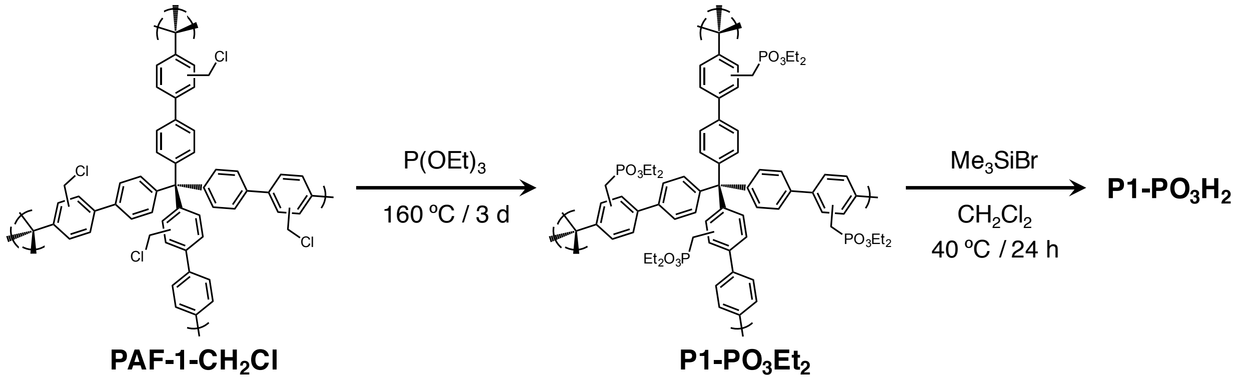

Scheme S3 Synthesis of polymers P1-PO_3Et_2 and P1-PO_3H_2

Synthesis of P1-PO_3Et_2: A 100-mL pressure tube was charged with PAF-1-CH_2Cl (200 mg) and subsequently neat triethyl phosphite ($P(OEt)_3$, 20 mL). The flask was sealed and heated at 160 ºC for 3 days. The resulting solid was collected by filtration and washed extensively with CH_2Cl_2, H_2O, MeOH, and THF. After drying the solid *in vacuo*, P1-PO_3Et_2 was obtained as a pale yellow powder (300 mg).

Synthesis of P1-PO_3H_2: An oven-dried 100-mL round bottomed flask charged with P1-PO_3Et_2 (300 mg) was sealed with a rubber septum, purged with argon, and then filled with 15 mL of anhydrous CH_2Cl_2. Bromotrimethylsilane (Me_3SiBr, 3 mL, 20 equiv. of phosphonate esters based on elemental analysis data) was added dropwise over 10 min. The resulting suspension was stirred at 40 ºC for 24 h, after which the mixture was filtered to remove solvent and unreacted Me_3SiBr, and then washed with CH_2Cl_2. The isolated solid was transferred into a 100-mL round bottomed flask again containing 50 mL of MeOH and was stirred for 6 h at room temperature. After removing solvent by filtration, the remaining solid, 10 mL of H_2O, and 10 mL of concentrated HCl was mixed in a flask and refluxed for 12 h in order to ensure the complete hydrolysis of phosphonate esters. The solid was collected by filtration, washed extensively with CH_2Cl_2, H_2O, MeOH, and THF, and then dried under reduced pressure to obtain P1-PO_3H_2 as a yellow powder (203 mg). Prior to gas adsorption measurements, the fully activated sample of P1-PO_3H_2 was obtained by heating at 110 ºC under vacuum.

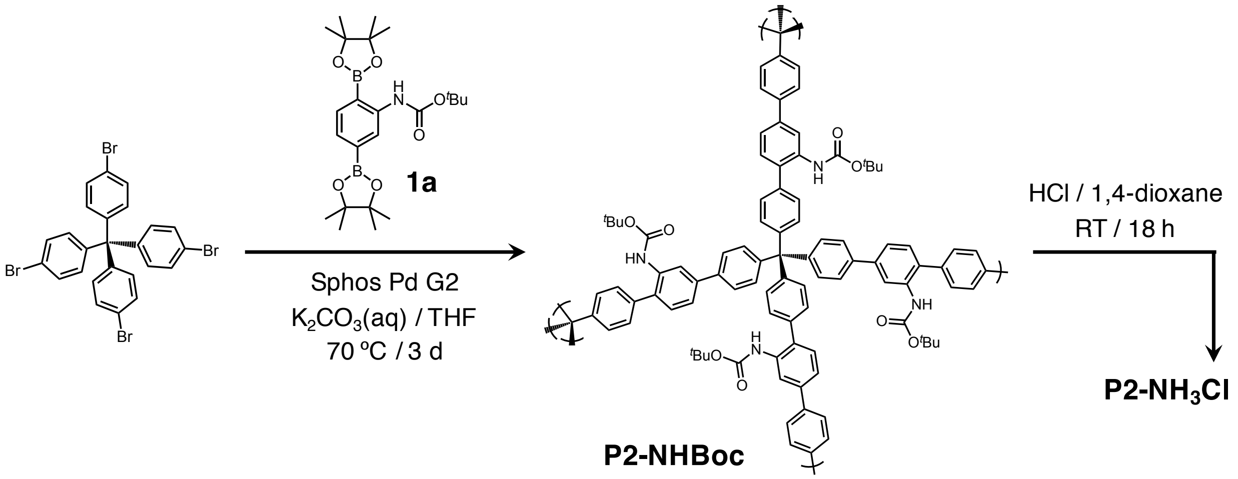

Scheme S4 Synthesis of polymers P2-NHBoc and P2-NH_3Cl

Synthesis of P2-NHBoc: Tetrakis(4-bromophenyl)methane (500 mg, 0.79 mmol), **1a** (770 mg, 1.73 mmol), and chloro(2-dicyclohexylphosphino-2',6'-dimethoxy-1,1'-biphenyl)[2-(2'-amino-1,1'-biphenyl)]palladium(II) (SPhos Pd G2) (45 mg, 0.062 mmol) were charged into a 100-mL 3-neck round bottomed flask, which was equipped with a reflux condenser and rubber stoppers. The solid mixture was subjected to three cycles of brief vacuum/argon (degas/backfill) with no stirring. Anhydrous THF (25 mL) and degassed aqueous K_2CO_3 (2 M, 2.5 mL) were transferred into the flask using a syringe under argon atmosphere and the solution was then stirred for 3 days at 70 ºC. The reaction became an extremely viscous gel during polymerization. The reaction mixture was cooled down to room temperature and insoluble product was isolated by filtration. Gel-like product turned into powder upon drying and was washed with THF, hot H_2O, hot EtOH, and hot $CHCl_3$. The product was further purified by Soxhlet extraction with THF for 24 h. The isolated solid was activated at 100 ºC under vacuum to yield P2-NHBoc as a beige powder (500 mg, 91%).

Synthesis of P2-NH_3Cl: P2-NHBoc (400 mg) was placed into a 100-mL round-bottomed flask containing 30 mL of HCl (4 N in 1,4-dioxane) solution. The suspension was stirred at room temperature for 18 h. The solid was isolated by filtration and washed extensively with 1,4,-dioxane and EtOH. The resulting powder was activated under vacuum at 100 ºC for 24 h, resulting in P2-NH_3Cl as a yellow/pale brown solid (300 mg).

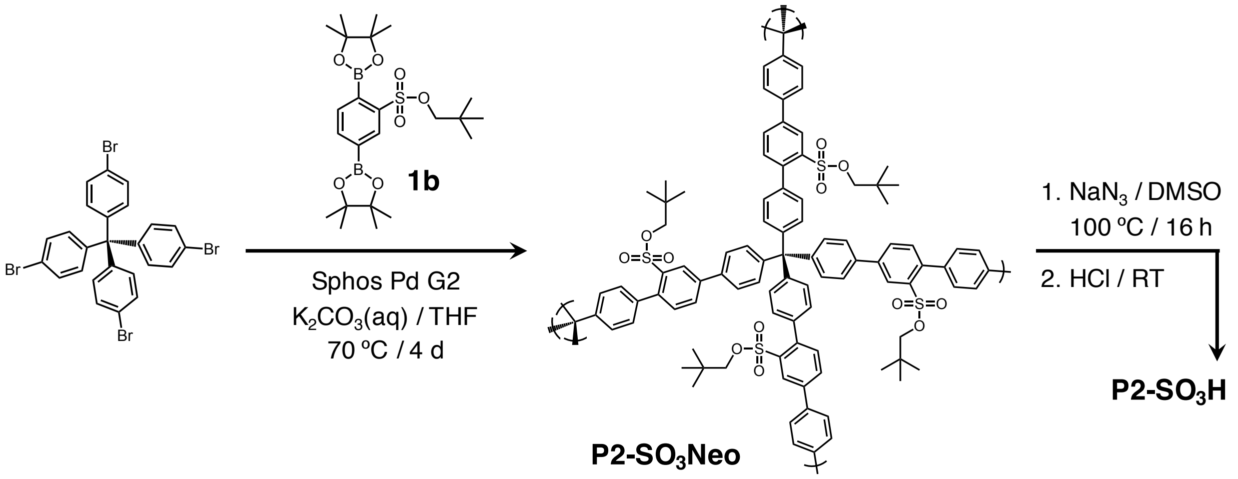

Scheme S5 Synthesis of polymers P2-SO_3Neo and P2-SO_3H

Synthesis of P2-SO_3Neo: Tetrakis(4-bromophenyl)methane (250 mg, 0.39 mmol), **1b** (400 mg, 0.83 mmol), and chloro(2-dicyclohexylphosphino-2’,6’-dimethoxy-1,1’-biphenyl)[2-(2’-amino-1,1’-biphenyl)]palladium(II) (SPhos Pd G2) (45 mg, 0.062 mmol) were charged into a 100-mL 3-neck round bottomed flask, which was equipped with a reflux condenser and rubber stoppers. The solid mixture was subjected to three cycles of brief vacuum/argon (degas/backfill) with no stirring. Anhydrous THF (25 mL) and degassed aqueous K_2CO_3 (2 M, 1.5 mL) were transferred into the flask using a syringe under argon atmosphere and the solution was then stirred for 4 days at 70 ºC. The reaction became an extremely viscous gel during polymerization. The reaction mixture was cooled down to room temperature and insoluble product was isolated by filtration. Gel-like product turned into powder upon drying and was washed with THF, hot H_2O, hot EtOH, and hot $CHCl_3$. The product was further purified by Soxhlet extraction with THF for 24 h. The isolated solid was activated at 80 ºC under vacuum to yield P2-SO_3Neo as a beige powder (290 mg, 95%).

Synthesis of P2-SO_3H: A100-mL round-bottomed flask was charged with P2-SO_3Neo (290 mg) followed by NaN_3 (730 mg) and DMSO (25 mL). The mixture was left to stir at 100 ºC for 16 h, after which the suspension was filtered. The isolated solid was subjected to same conditions with fresh NaN_3/DMSO for another 16 h. After filtering, the solid was washed with copious amount of H_2O and suspended in an HCl solution (3 M, 50 mL) at room temperature for 8 h. The solvent was decanted, replaced with 6 M HCl solution (50 mL), and stirred overnight at room temperature. The polymer was filtered and washed extensively with H_2O, MeOH, and THF. Finally, it was further purified by Soxhlet extraction with THF for 24 h, which yielded P2-SO_3H as a yellow powder (230 mg). Prior to gas adsorption measurements, the fully activated sample of P2-SO_3H was obtained by heating at 80 ºC under vacuum.

3. Structural Characterization of Polymers

3.1. Elemental Analysis

Table S1 Elemental analysis data of P1 polymers and their intermediates

	**PAF-1**		**P1-NH_3Cl**		**P1-SO_3H**		**PAF-1-CH_2Cl**		**P1-PO_3Et_2**		**P1-PO_3H_2**	
%	Calcd.	Found	Calcd.[a]	Found	Calcd.[a]	Found	Calcd.[a]	Found	Calcd.[b]	Found	Calcd.[c]	Found
C	94.90	91.68	71.61	75.98	63.01	61.68	78.46	75.77	69.26	65.09	65.79	55.37
H	5.10	5.26	4.81	4.89	3.38	3.46	4.39	4.53	6.14	6.77	4.40	4.98
N	0.00	0.59	6.68	8.33	0.00	0.39	0.00	0.35	0.00	0.25	0.00	0.26
S					13.46	11.99						
Cl			16.90	*			17.15	16.15	0.00	0.59		
P									9.65	8.78	11.69	9.86
O					20.15	*			14.95	*	18.12	*

*Not measured. [a] Assuming the installation of one functional group per biphenyl linker. [b] Assuming the complete conversion of –CH_2Cl to –$CH_2PO_3Et_2$ based on the experimentally determined chlorine content of PAF-1-CH_2Cl. [c] Assuming the complete hydrolysis of phosphonate esters of P1-PO_3Et_2 based on its calculated values.

Table S2 Elemental analysis data of P2 polymers

	**P2-NHBoc**		**P2-NH_3Cl**		**P2-SO_3Neo**		**P2-SO_3H**		**P2-$CO_2C_9H_{19}$**		**P2-CO_2H**	
%	Calcd.[a]	Found	Calcd.[b]	Found	Calcd.[a]	Found	Calcd.[b]	Found	Calcd.[a]	Found	Calcd.[b]	Found
C	80.78	75.08	77.76	79.70	73.41	69.40	70.69	61.92	80.45	76.58	76.39	66.07
H	6.06	6.18	4.94	5.33	5.77	5.89	3.85	4.94	8.42	7.91	3.75	4.17
N	4.01	3.99	4.90	4.79	0.00	0.00	0.00	0.00	0.00	0.00	0.00	0.00
S					8.34	8.00	10.20	8.95				
Cl			12.40	*								
O	9.15	*			12.48	*	15.26	*	11.13	11.75	19.86	*

*Not measured. [a] Calculated for ideal polymerization. [b] Calculated for the complete removal of protection groups of corresponding ideal polymers.

3.2. FTIR Spectroscopy

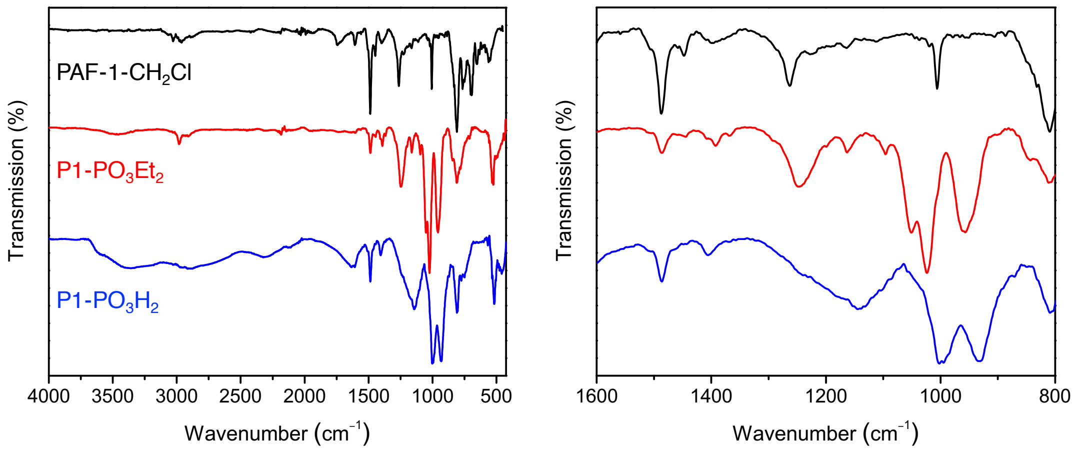

Fig. S7 FTIR-ATR spectra of PAF-1-CH_2Cl, P1-PO_3Et_2, and P1-PO_3H_2.

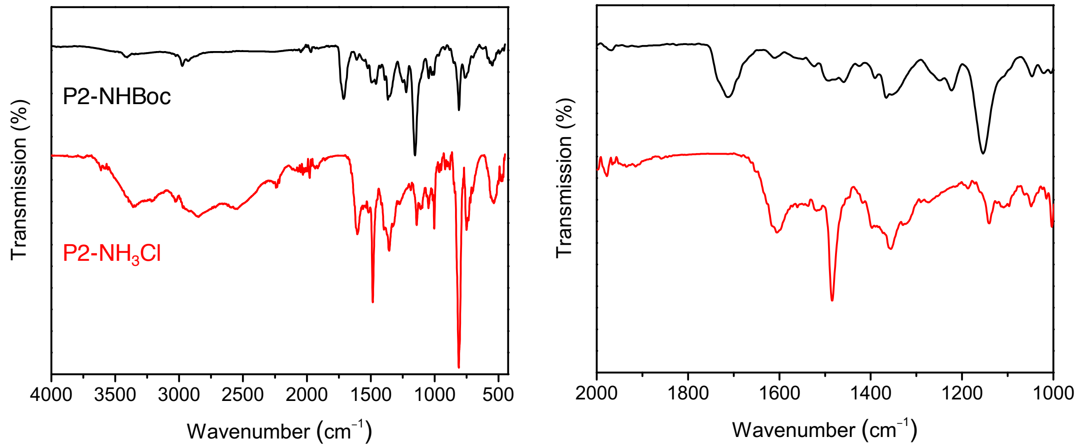

Fig. S8 FTIR-ATR spectra of P2-NHBoc and P2-NH_3Cl.

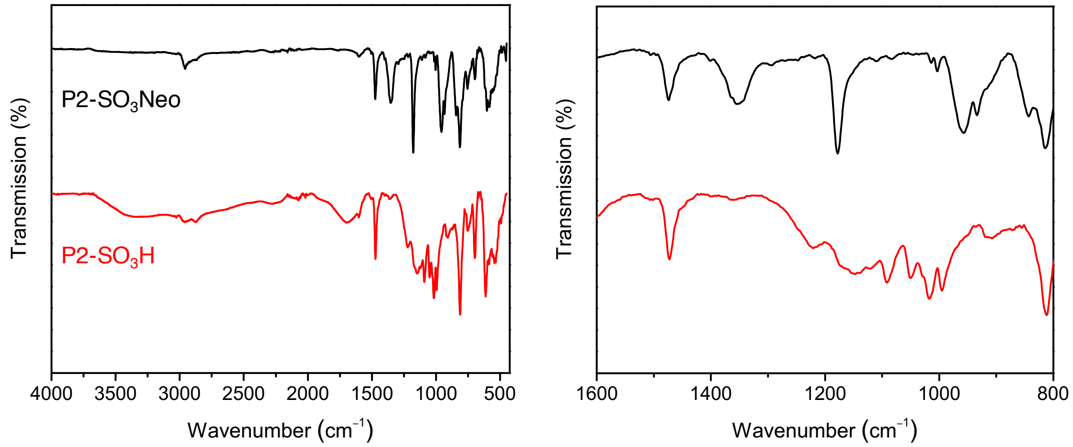

Fig. S9 FTIR-ATR spectra of P2-SO_3Neo and P2-SO_3H.

3.3. Solid-State NMR Spectroscopy

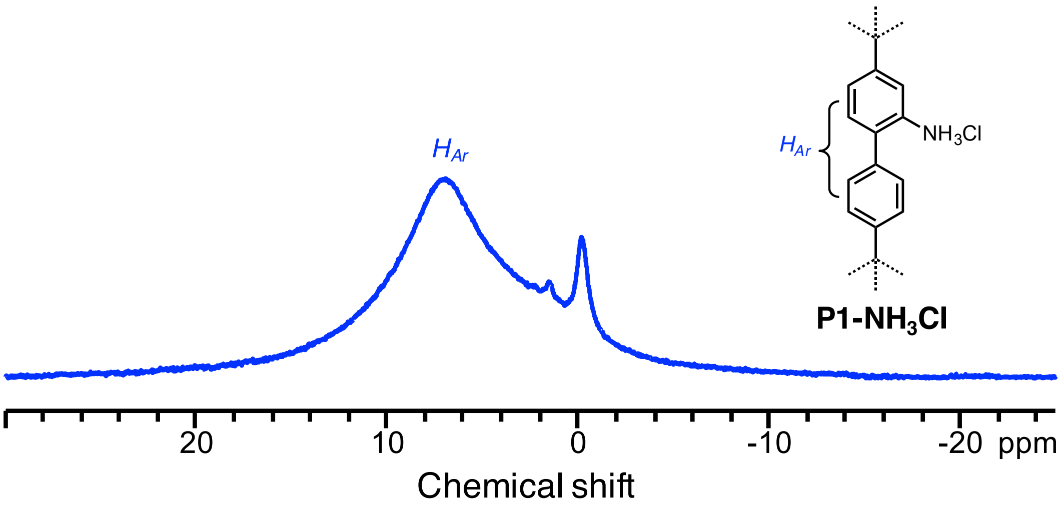

Fig. S10 Solid-state MAS ^{1}H NMR spectrum of P1-NH_3Cl. The chemical shift assignment for aromatic protons is in agreement with the previously reported data in the literature.[4]

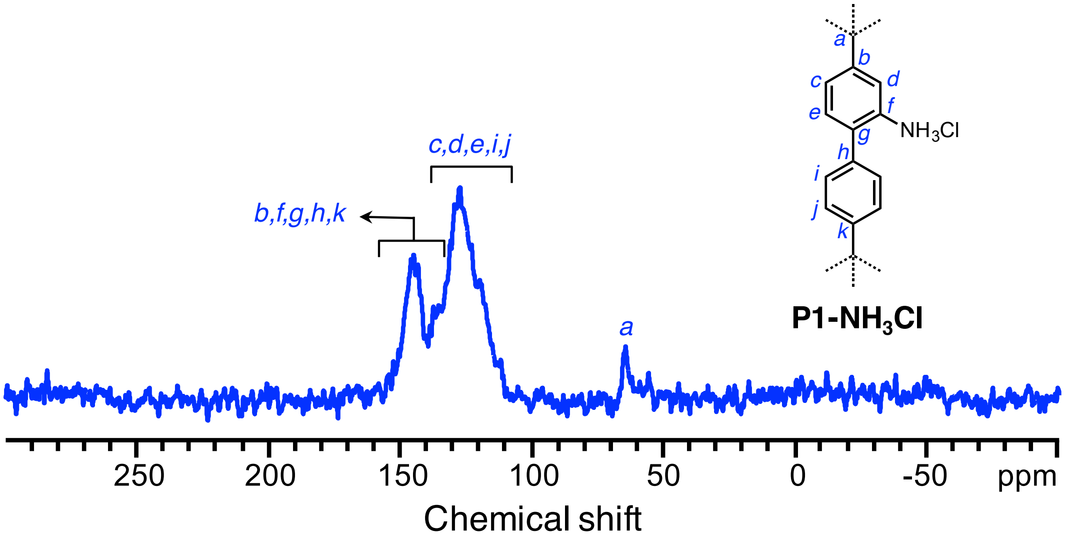

Fig. S11 Solid-state CP/MAS ^{13}C NMR spectrum of P1-NH_3Cl. The chemical shift assignments are in agreement with the previously reported data in the literature.[9-11]

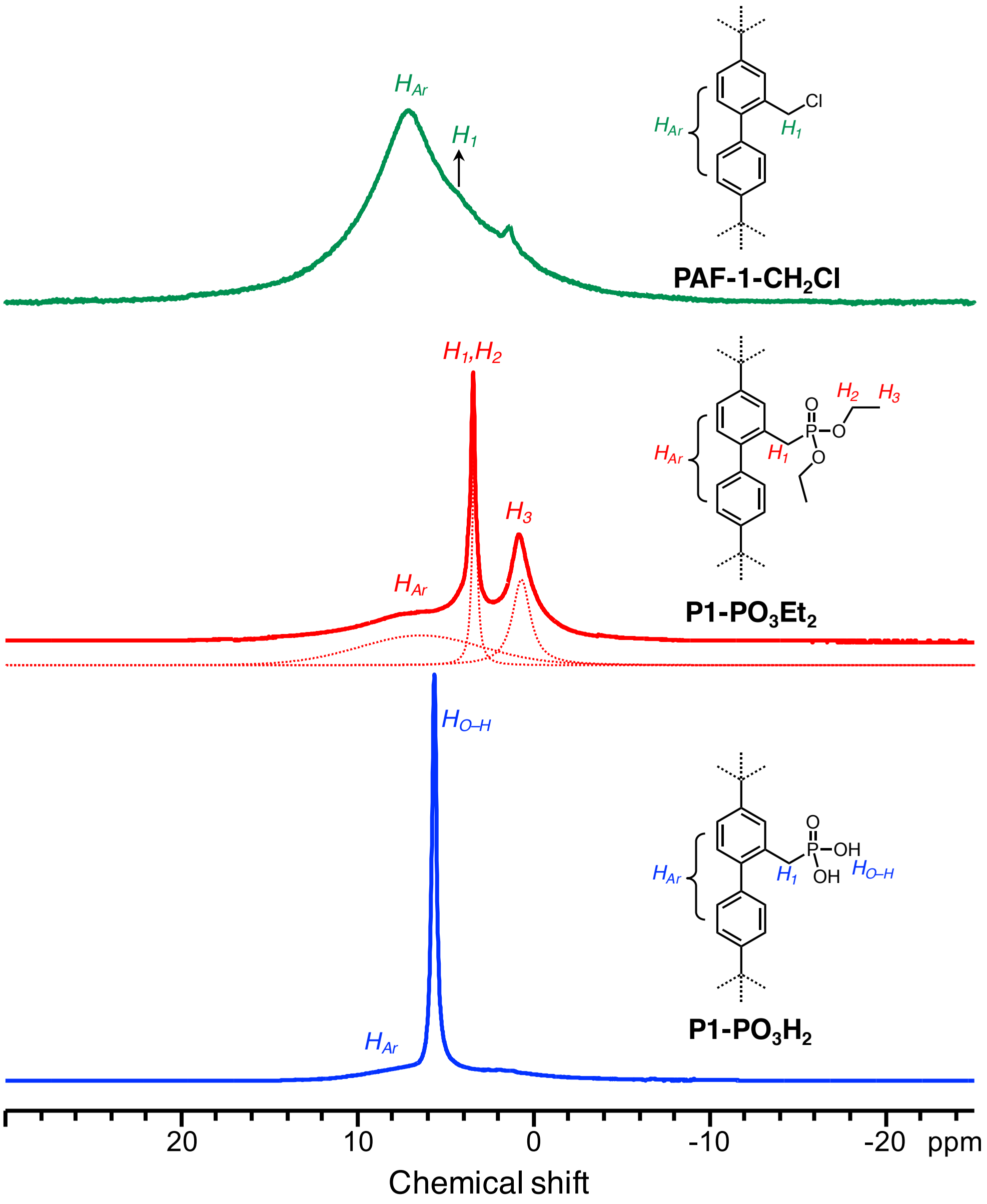

Fig. S12 Solid-state MAS ^{1}H NMR spectra of PAF-1-CH_2Cl (green), P1-PO_3Et_2 (red) and P1-PO_3H_2 (blue). The chemical shift assignments are in agreement with the previously reported data in the literature.[4,12] Dotted lines (red) represent deconvoluted peaks for P1-PO_3Et_2, in which the chemical shifts at 0.8 and 3.4 ppm were assigned to H_3 and H_1–H_2, respectively. The peak associated with H_1 in P1-PO_3H_2 (blue) is suppressed by the H_{O-H} peak.

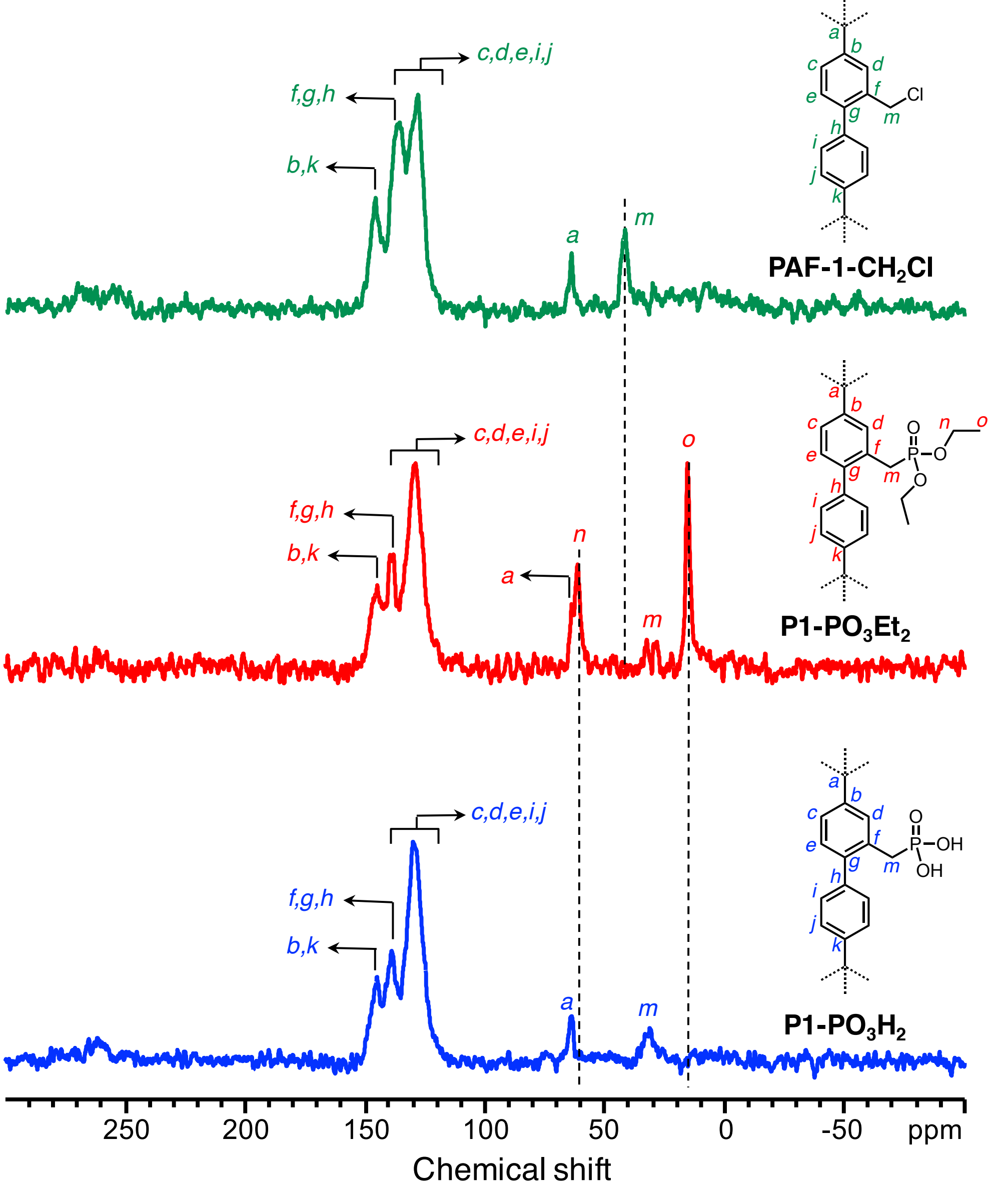

Fig. S13 Solid-state CP/MAS ^{13}C NMR spectra of PAF-1-CH_2Cl (green), P1-PO_3Et_2 (red) and P1-PO_3H_2 (blue). The chemical shift assignments are in agreement with the previously reported data in the literature.[9-10,13] The vertical dashed lines represent the disappearance of related chemical shifts upon functionalization of the polymer.

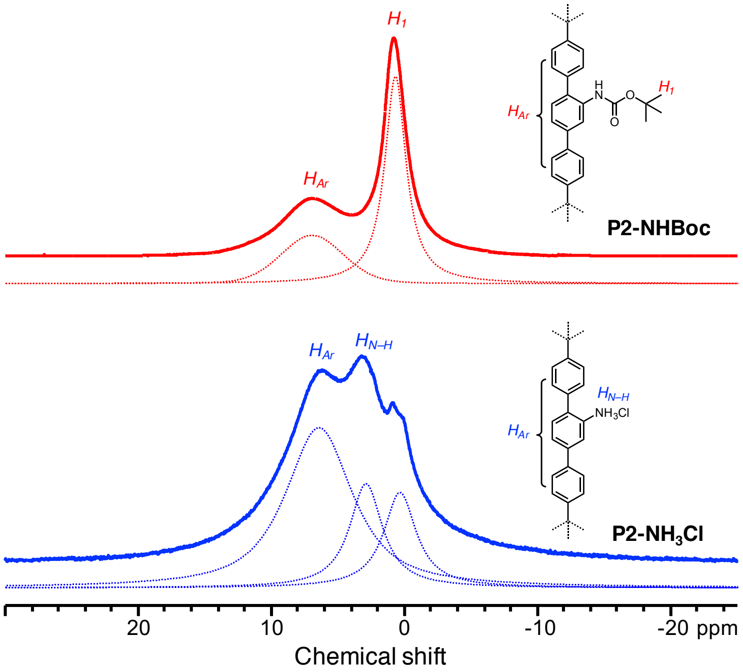

Fig. S14 Solid-state MAS ^{1}H NMR spectra of P2-NHBoc (red) and P2-NH_3Cl (blue). The chemical shift assignments are in agreement with the previously reported data in the literature.[2,4] Dotted lines represent deconvoluted peaks. The peak at 0.8 ppm in P2-NHBoc (red) corresponds to the t-butyl protons, which disappears upon its conversion to P2-NH_3Cl. The chemical shift at 3.0 ppm in the deconvoluted spectrum P2-NH_3Cl (blue) is assigned to N–H protons.

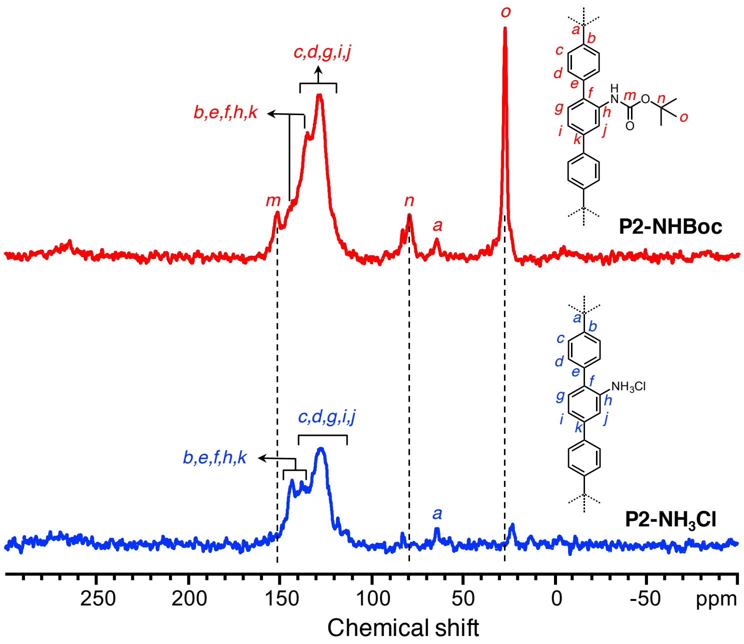

Fig. S15 Solid-state CP/MAS ^{13}C NMR spectra of P2-NHBoc (red) and P2-NH_3Cl (blue). The chemical shift assignments are in agreement with the previously reported data in the literature.[2,10-11] The vertical dashed lines represent the changes in the spectrum upon removal of Boc groups.

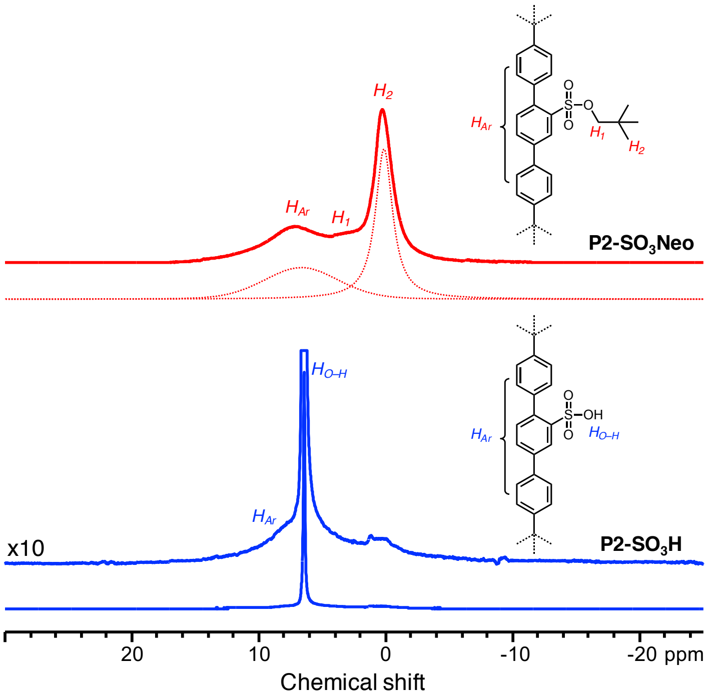

Fig. S16 Solid-state MAS ^{1}H NMR spectra of P2-SO$_3$Neo (red) and P2-SO$_3$H (blue). The chemical shift assignments are in agreement with the previously reported data in the literature.[4,8] Dotted lines represent deconvoluted peaks. The chemical shift corresponding to H_2 protons at 0.2 ppm in P2-SO$_3$Neo disappears upon hydrolysis to P2-SO$_3$H.

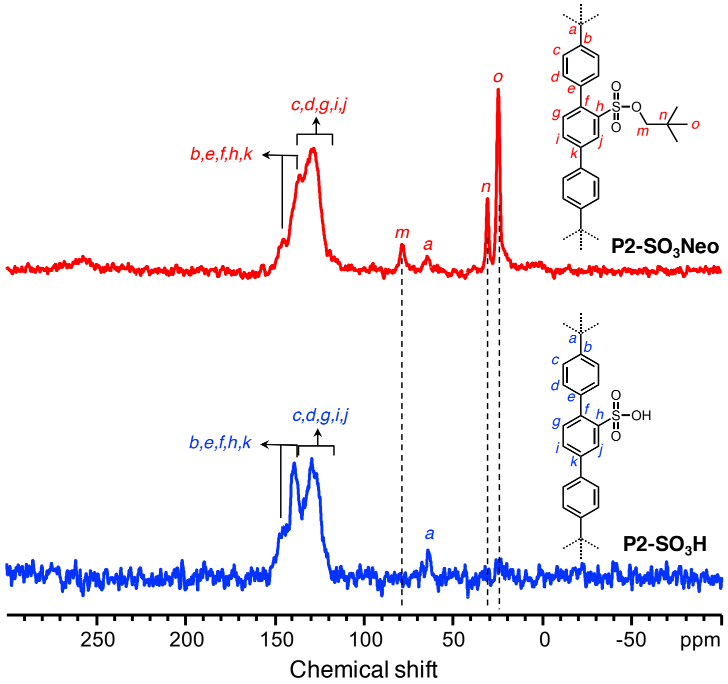

Fig. S17 Solid-state CP/MAS ^{13}C NMR spectra of P2-SO$_3$Neo (red) and P2-SO$_3$H (blue). The chemical shift assignments are in agreement with the previously reported data in the literature.[8,11] The vertical dashed lines indicate the removal of carbon atoms associated with the neopentyl group.

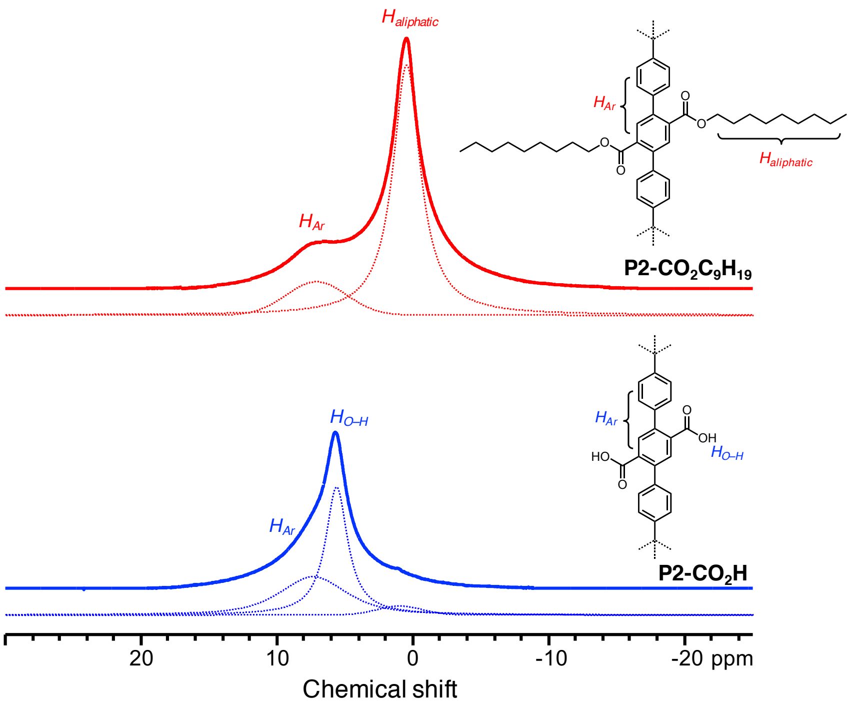

Fig. S18 Solid-state MAS ^{1}H NMR spectra of P2-$CO_2C_9H_{19}$ (red) and P2-CO_2H (blue). The chemical shift assignments are in agreement with the previously reported data in the literature.[4,14] Dotted lines represent deconvoluted peaks, which revealed the presence of aromatic (7.2 ppm) and aliphatic (0.5 ppm) protons in the case of P2-$CO_2C_9H_{19}$ as well as the disappearance of aliphatic protons in P2-CO_2H.

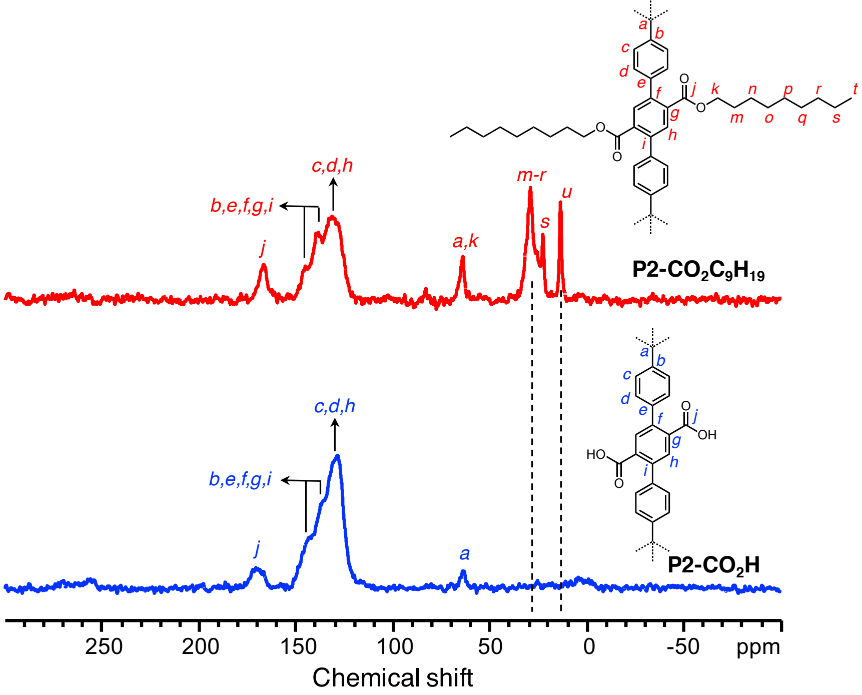

Fig. S19 Solid-state CP/MAS ^{13}C NMR spectra of P2-$CO_2C_9H_{19}$ (red) and P2-CO_2H (blue). The chemical shift assignments are in agreement with the previously reported data in the literature.[14-15] The vertical dashed lines indicate the complete removal of aliphatic side chains upon hydrolysis.

3.4. Scanning Electron Microscopy

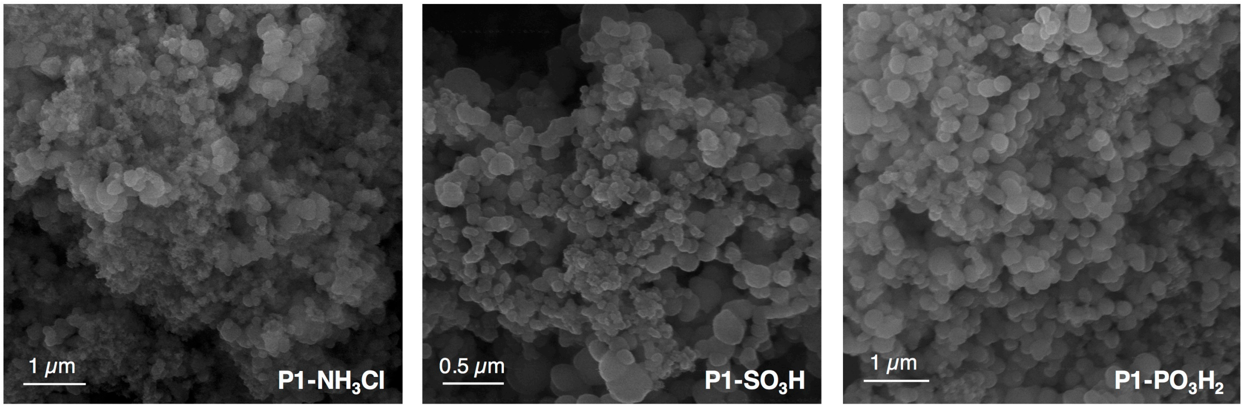

Fig. S20 SEM images of P1-NH_3Cl, P1-SO_3H, and P1-PO_3H_2.

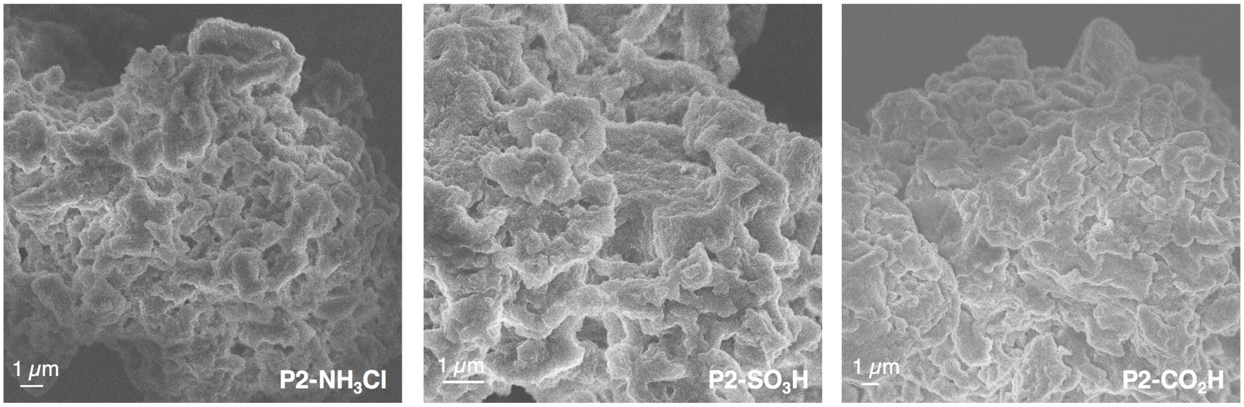

Fig. S21 SEM images of P2-NH_3Cl, P2-SO_3H, and P2-CO_2H.

3.5. Thermogravimetric Analysis

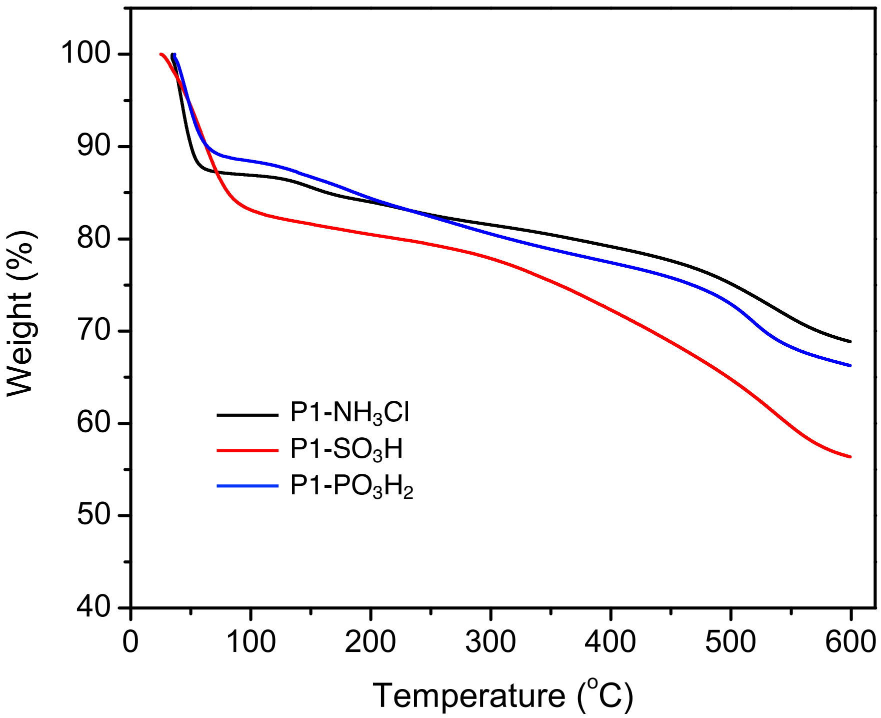

Fig. S22 Thermogravimetric analysis of P1-NH_3Cl, P1-SO_3H, and P1-PO_3H_2.

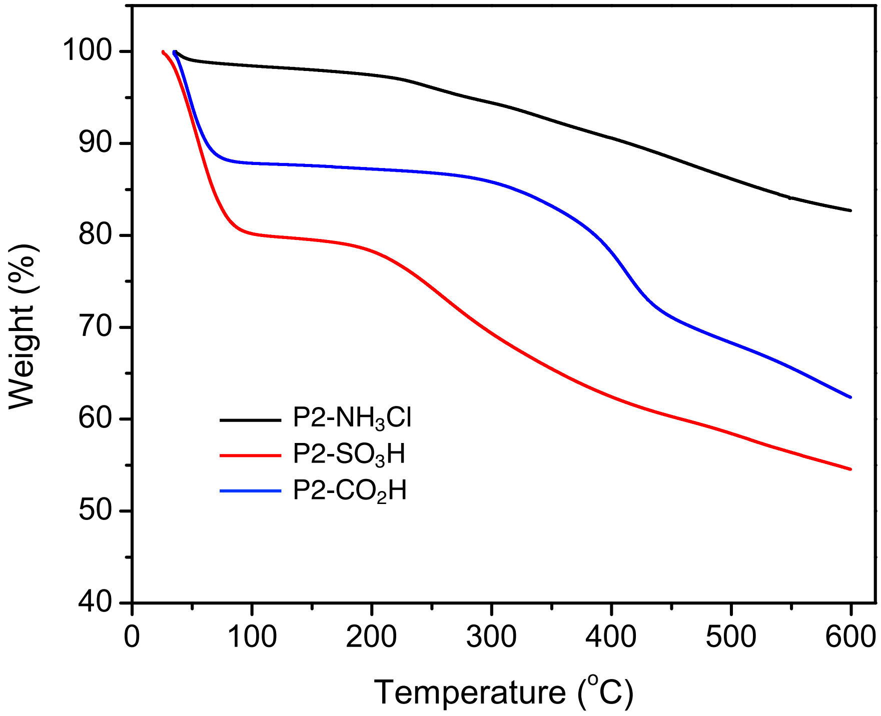

Fig. S23 Thermogravimetric analysis of P2-NH_3Cl, P2-SO_3H, and P2-CO_2H.

3.6. Surface Area and Pore Size Distributions

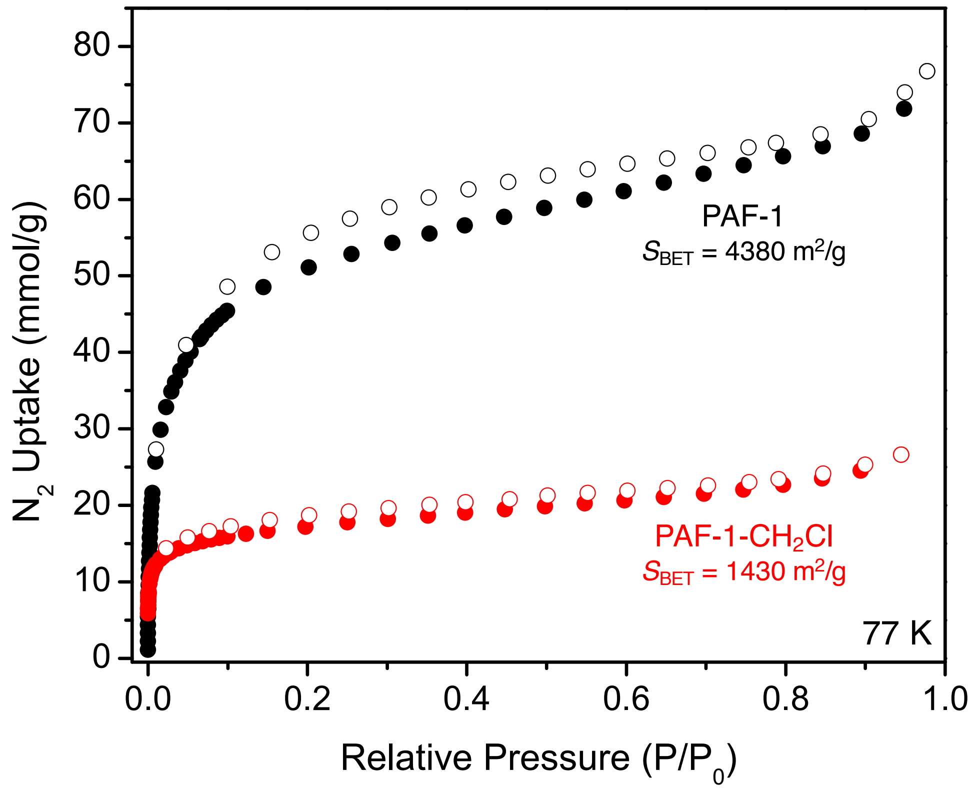

Fig. S24 Nitrogen adsorption isotherms of PAF-1 and PAF-1-CH_2Cl used in the synthesis of P1 polymers. Closed and open symbols represent adsorption and desorption branches, respectively.

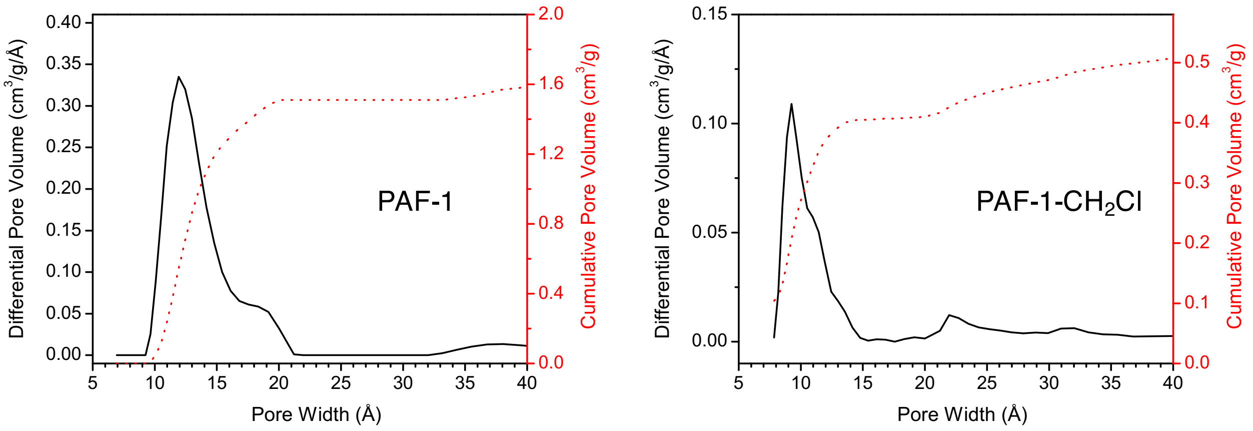

Fig. S25 Pore size distributions of PAF-1 and PAF-1-CH_2Cl.

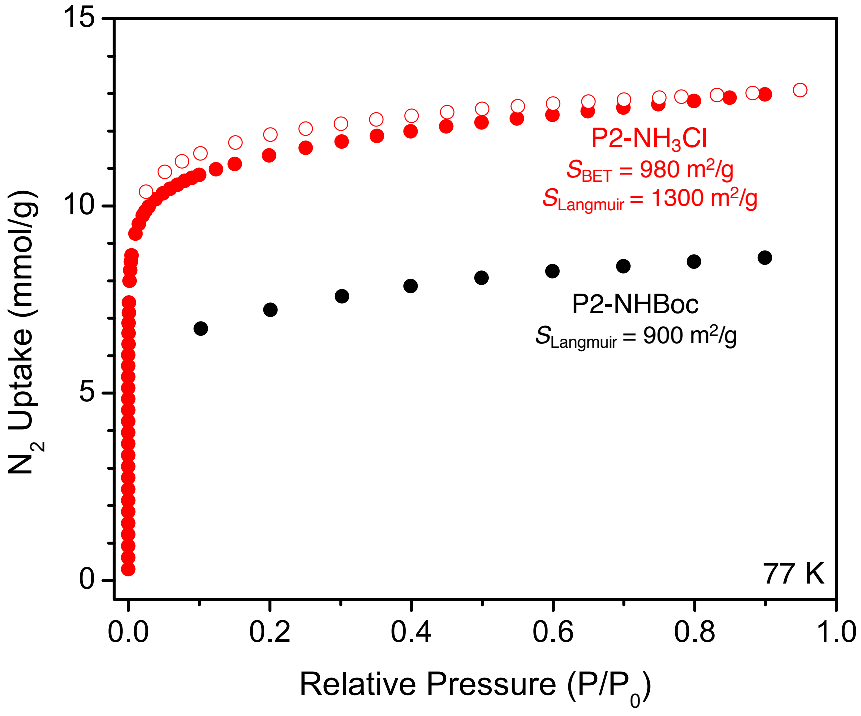

Fig. S26 Nitrogen adsorption isotherms of P2-NHBoc (before deprotection) and P2-NH_3Cl (after deprotection). Closed and open symbols represent adsorption and desorption branches, respectively.

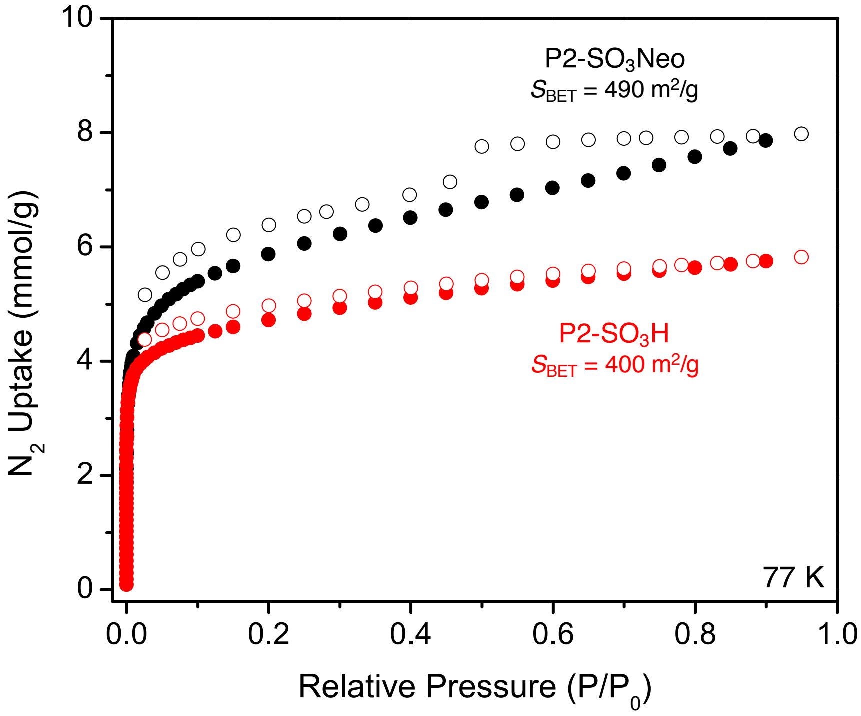

Fig. S27 Nitrogen adsorption isotherms of P2-SO_3Neo (before deprotection) and P2-SO_3H (after deprotection). Closed and open symbols represent adsorption and desorption branches, respectively.

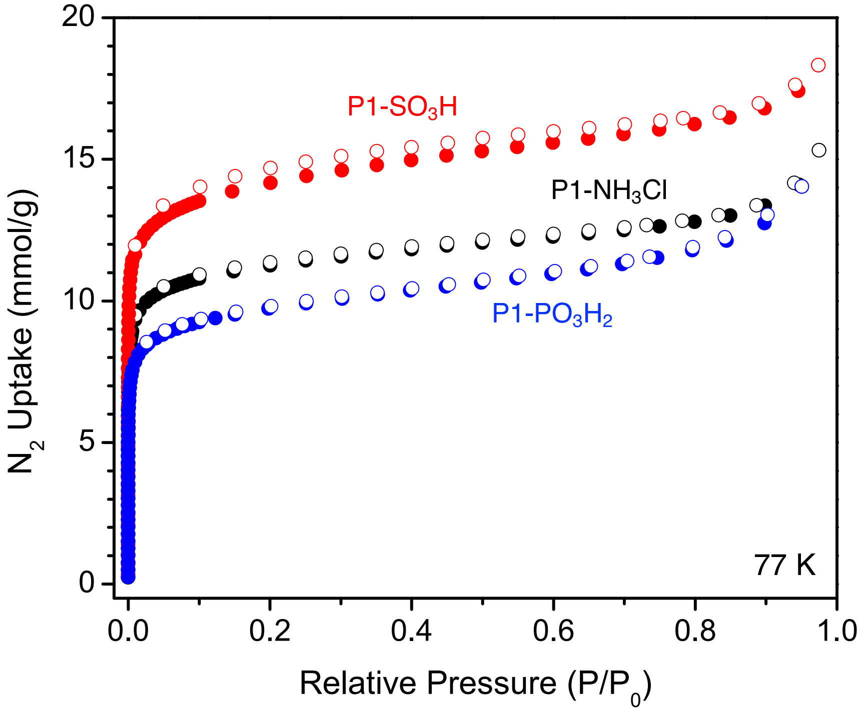

Fig. S28 Nitrogen adsorption isotherms of P1-NH_3Cl, P1-SO_3H, and P1-PO_3H_2. Closed and open symbols represent adsorption and desorption branches, respectively.

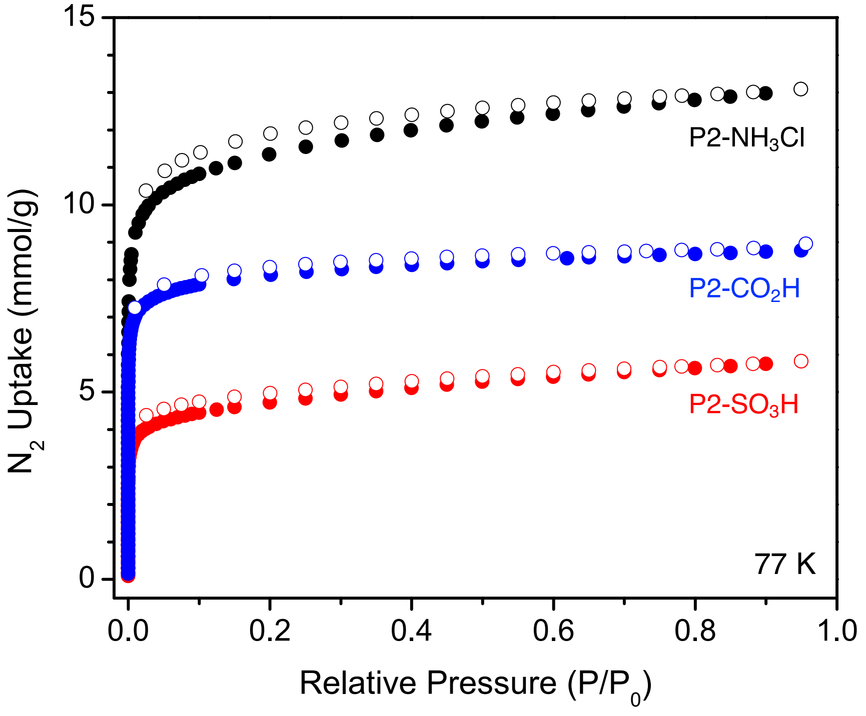

Fig. S29 Nitrogen adsorption isotherms of P2-NH_3Cl, P2-SO_3H, and P2-CO_2H. Closed and open symbols represent adsorption and desorption branches, respectively.

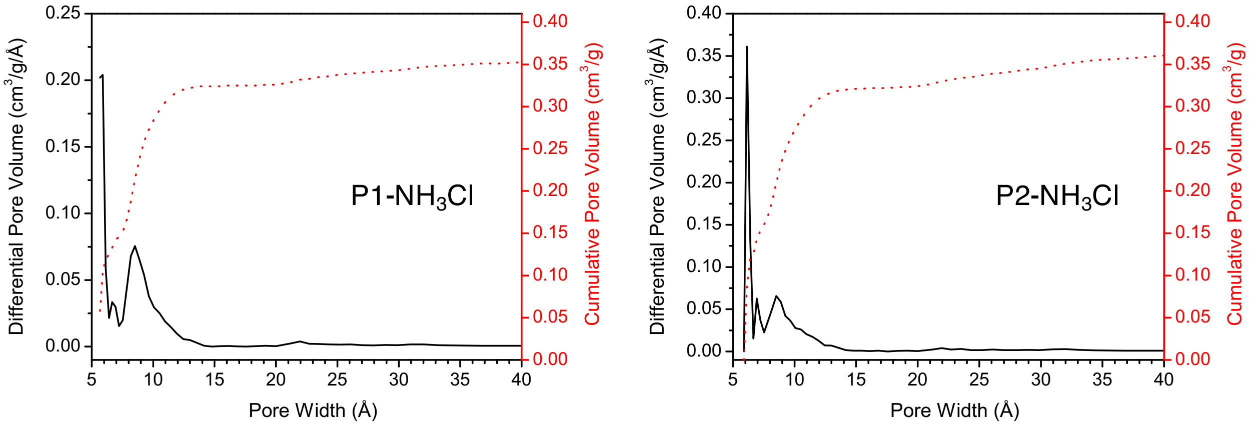

Fig. S30 Pore size distributions of P1-NH_3Cl and P2-NH_3Cl.

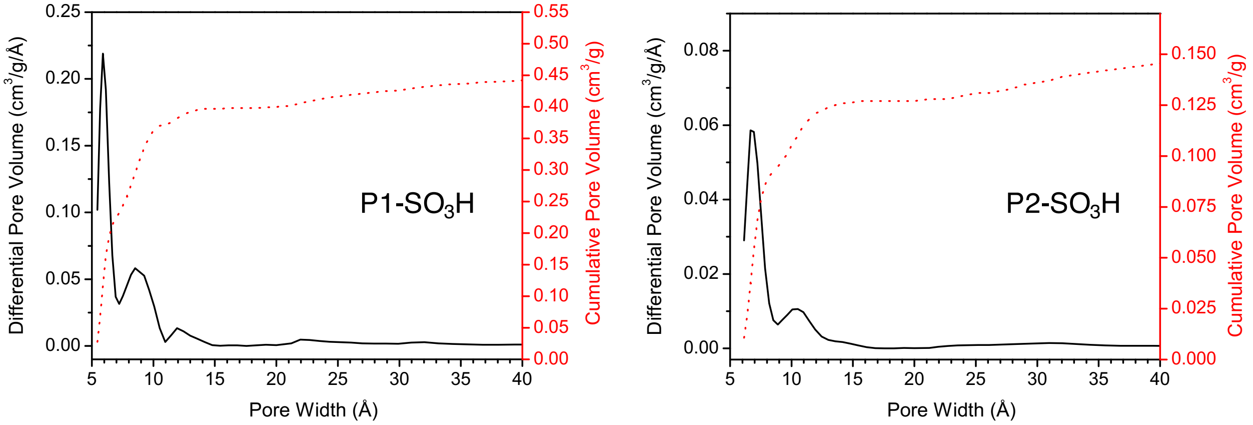

Fig. S31 Pore size distributions of P1-SO_3H and P2-SO_3H.

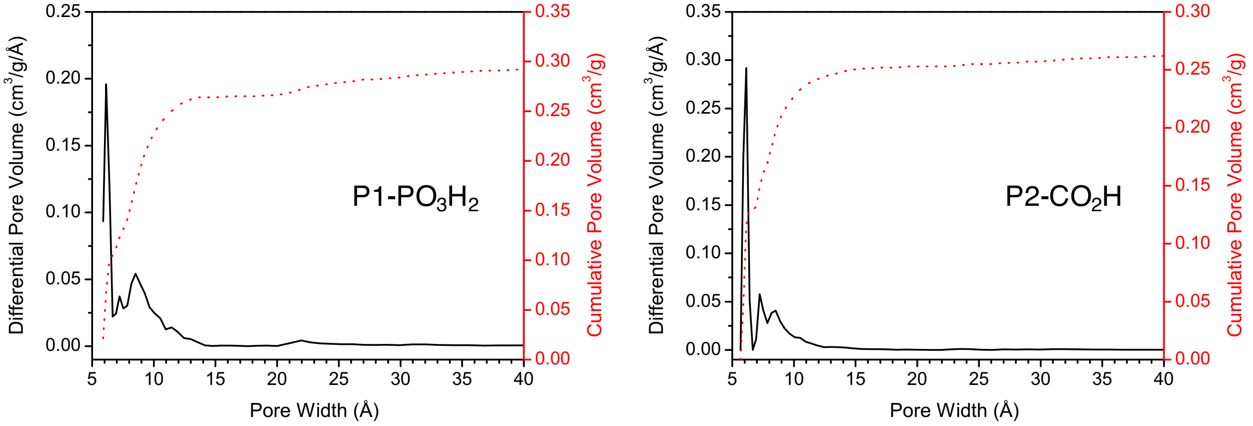

Fig. S32 Pore size distributions of P1-PO_3H_2 and P2-CO_2H.

4. NH_3 Adsorption

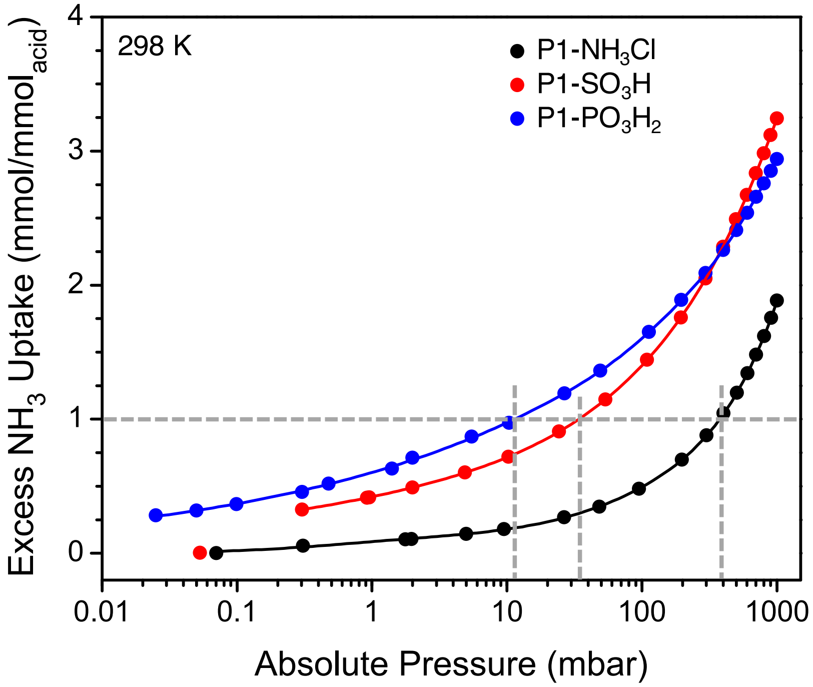

Fig. S33 NH_3 isotherms of P1 polymers plotted with respect to the density of acidic sites. The intersection of dashed gray lines demonstrates pressure points where one ammonia per acid is achieved.

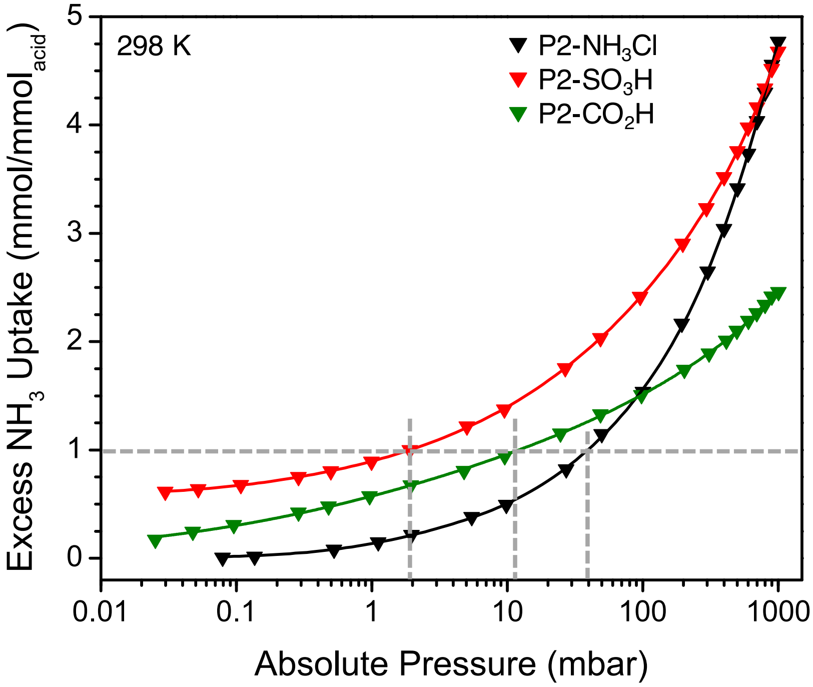

Fig. S34 NH_3 isotherms of P2 polymers plotted with respect to the density of acidic sites. The intersection of dashed gray lines demonstrates pressure points where one ammonia per acid is achieved.

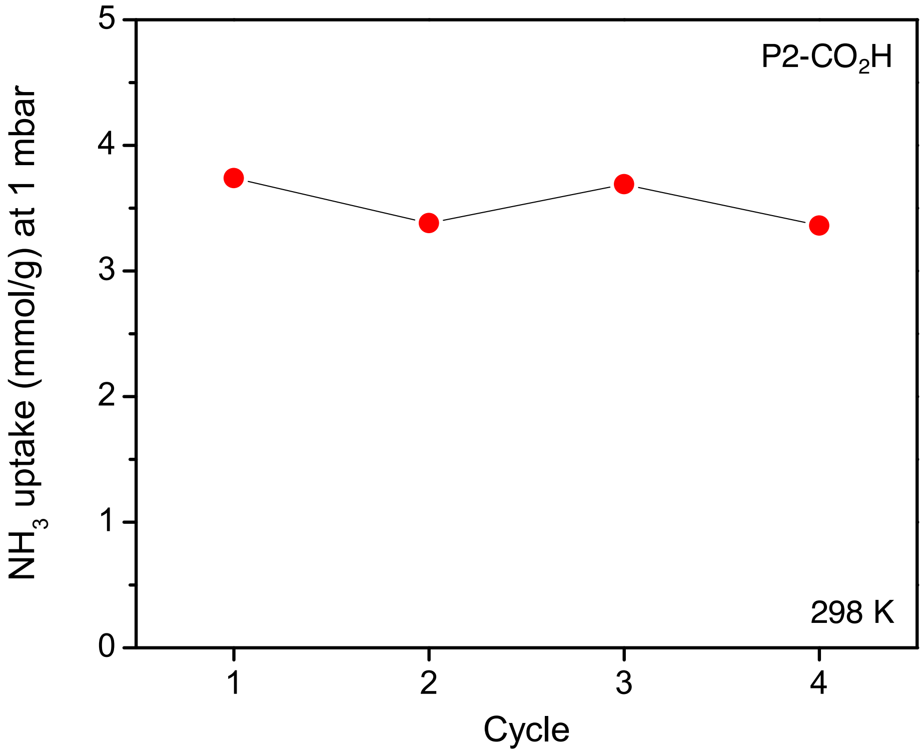

Fig. S35 Cycling experiments were performed on P2-CO_2H and NH_3 uptake capacity at 1 mbar was measured in each cycle. The sample was reactivated at 130 °C for 12 h between each cycles.

5. Breakthrough Experiments

Breakthrough testing was conducted on porous polymers using a microbreakthrough setup (Fig. S36) that has been described previously.[16-18] Briefly, neat ammonia was injected into a steel canister, which was then pressurized to approximately 15 psig. A stream from this ballast was delivered via mass flow controller and mixed with humidity-controlled stream at a rate necessary to achieve 2000 mg/m^3. The mixed stream was delivered at a total flow rate of 20 mL/min to a glass-fritted tube submerged in a temperature-controlled bath at 20 °C. Within the 4 mm ID tube, polymers were packed to a bed depth of approximately 4 mm, resulting in a residence time of approximately 0.15 s. Breakthrough was measured on the effluent side of the bed using HP5890 Series II gas chromatographs equipped with a photoionization detector. The effluent curve was integrated to calculate the loading of ammonia.

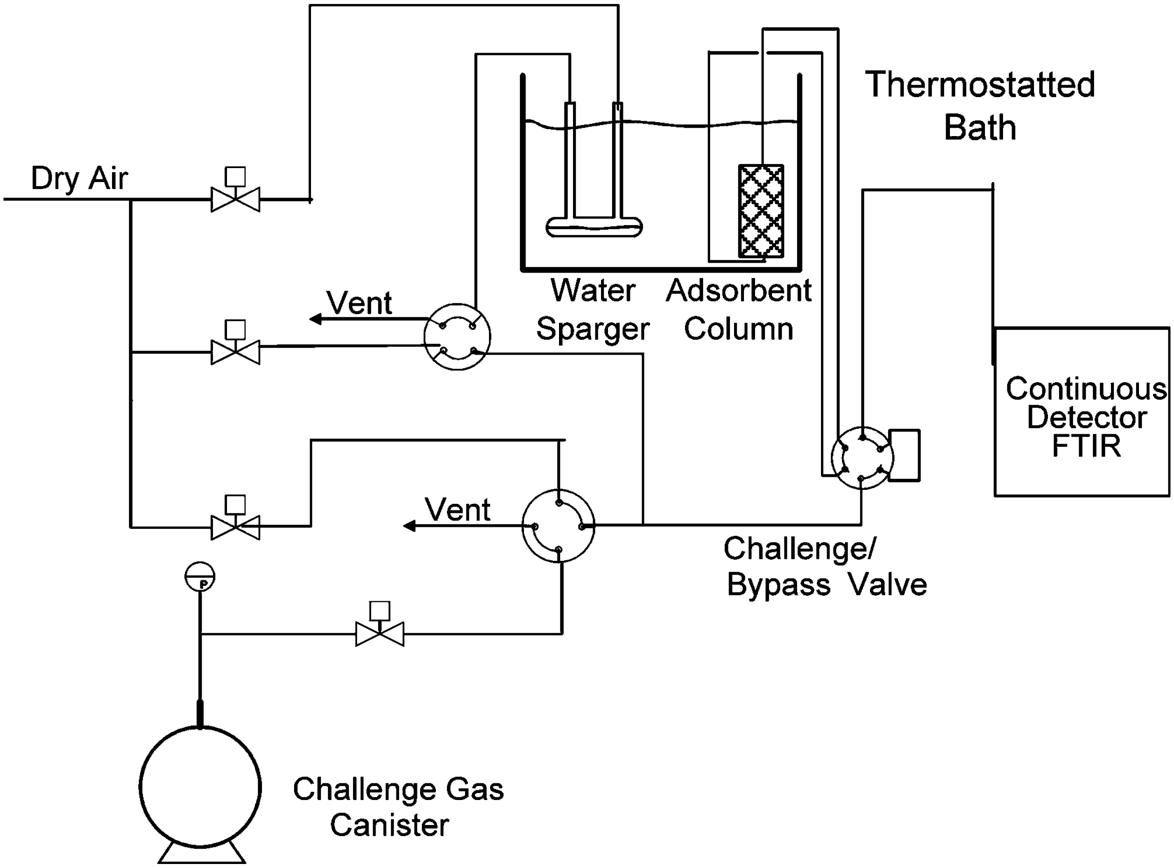

Fig. S36 Microbreakthrough system.

6. *In Situ* Infrared Spectroscopy

FTIR spectra were collected at 2 cm^{-1} resolution on a Bruker Vertex 70 spectrophotometer, equipped with a MCT cryodetector, at “beam temperature”—i.e., the temperature reached by samples under the IR beam. The samples were examined in the form of self-supporting pellets mechanically protected with a pure gold frame (P1-SO_3H and P1-PO_3H_2) or in the form of thin layer depositions on Si wafers, starting from aqueous suspensions (PAF-1 and P2-CO_2H). Before NH_3 adsorption, all samples were activated in controlled atmosphere, at the corresponding activation temperature, using a home-made quartz IR cell equipped with KBr windows and characterized by a small optical path (2 mm). The cell was connected to a conventional high-vacuum glass line, equipped with mechanical and turbo molecular pumps (capable of a residual pressure $p < 10^{-4}$ mbar), which allows performing *in situ* adsorption/desorption experiments of molecular probes.

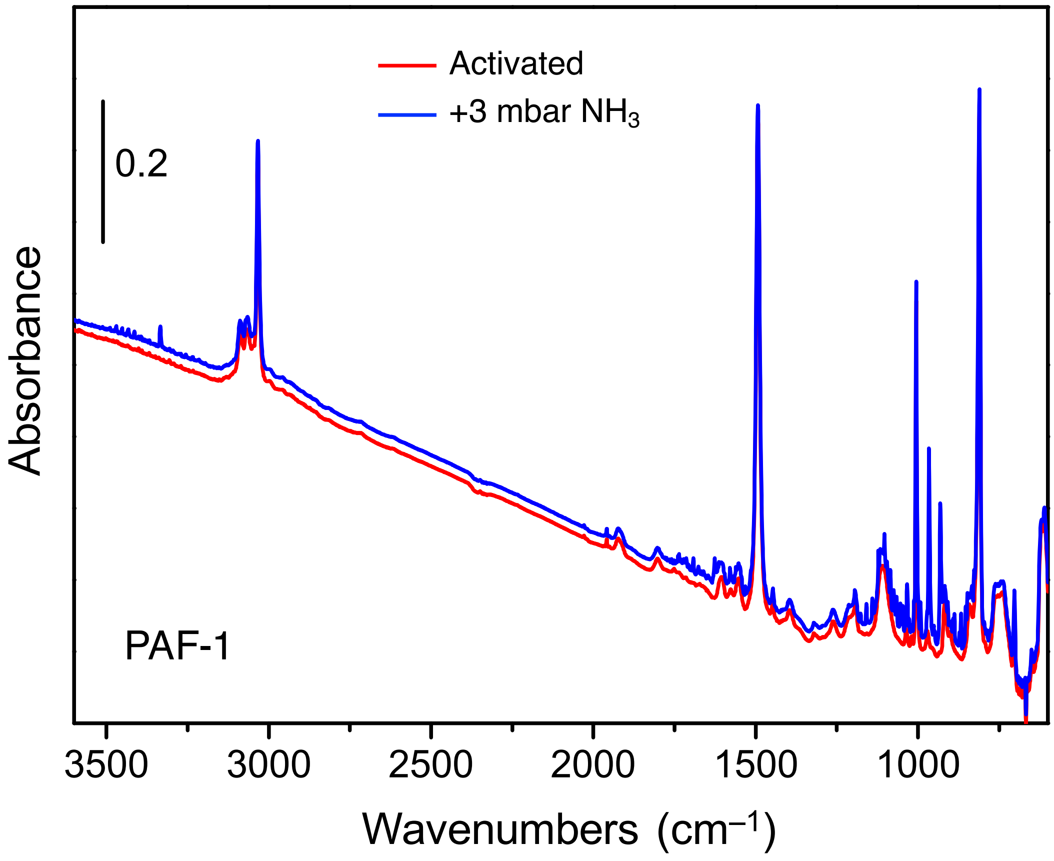

Fig. S37 Infrared spectra of PAF-1 collected at 298 K after its thermal activation under vacuum (red line) and equilibration with ammonia at an equilibrium pressure of 3 mbar (blue line).

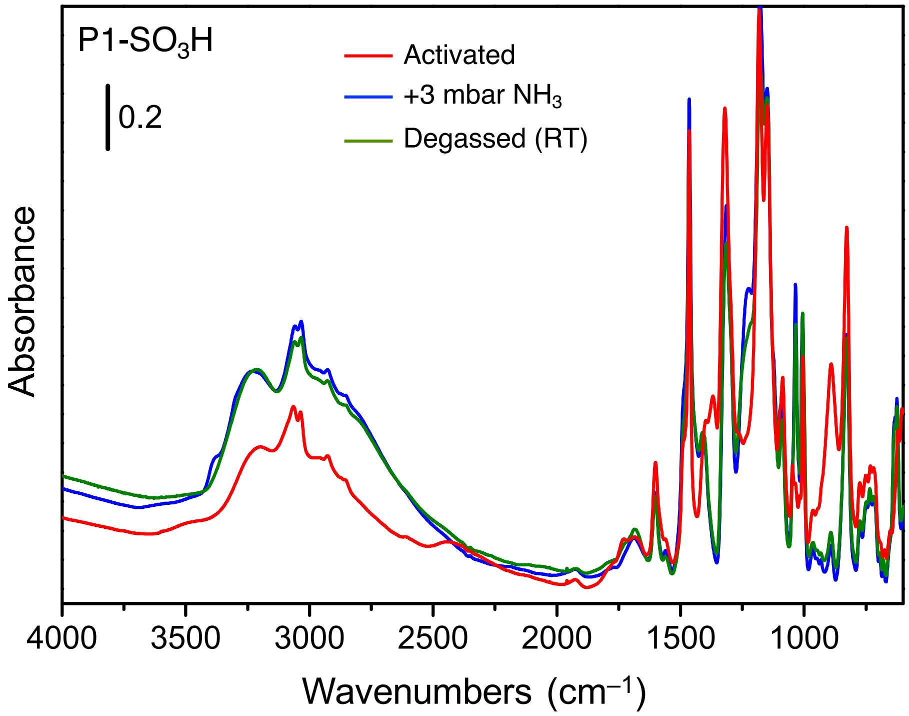

Fig. S38 Infrared spectra of P1-SO$_3$H collected at 298 K after its thermal activation under vacuum (red line), equilibration with ammonia at an equilibrium pressure of 3 mbar (blue line), and evacuation under vacuum (green line).

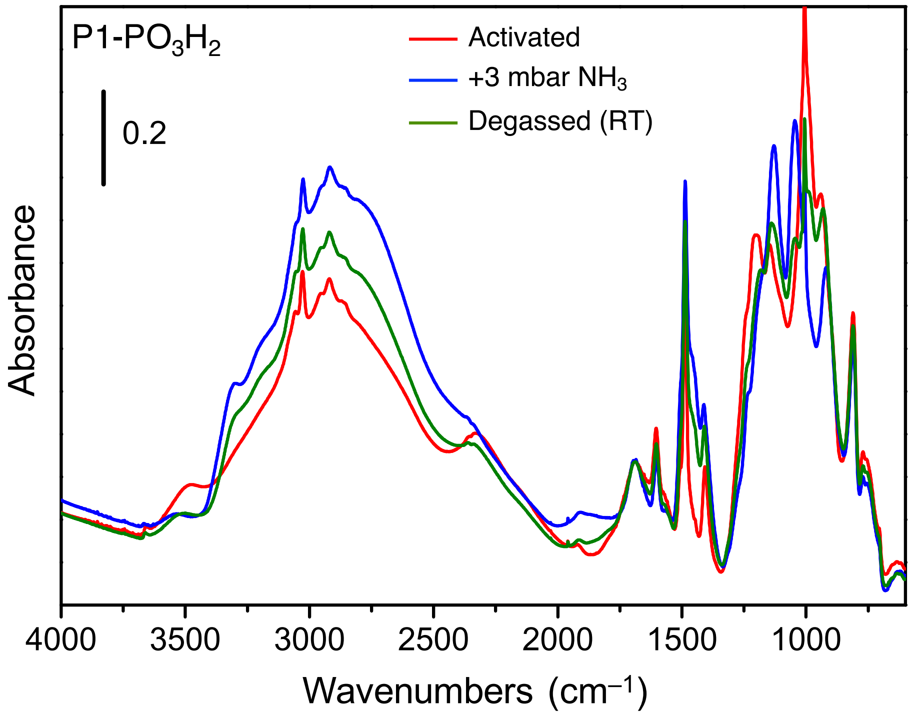

Fig. S39 Infrared spectra of P1-PO$_3$H$_2$ collected at 298 K after its thermal activation under vacuum (red line), equilibration with ammonia at an equilibrium pressure of 3 mbar (blue line), and evacuation under vacuum (green line).

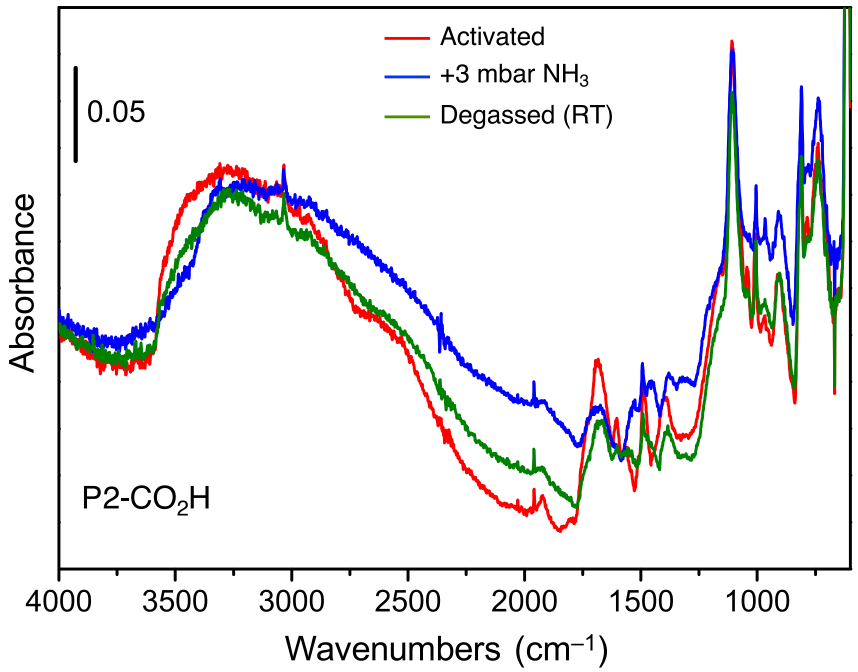

Fig. S40 Infrared spectra of P2-CO_2H collected at 298 K after its thermal activation under vacuum (red line), equilibration with ammonia at an equilibrium pressure of 3 mbar (blue line), and evacuation under vacuum (green line).

7. Water Isotherm for P1-SO_3H

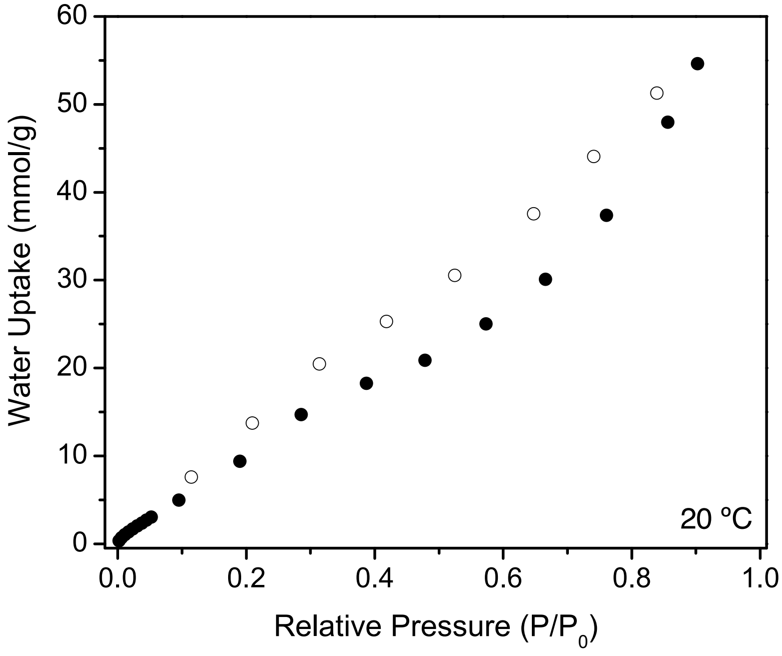

Fig. S41 Water adsorption isotherm of P1-SO_3H at 20 ºC (P_0 = 23.393 mbar). Closed and open symbols represent adsorption and desorption branches, respectively.

8. List of Porous Materials and Their NH_3 Uptake Capacities

Table S3 Low-pressure NH_3 uptake capacities of metal-organic frameworks and porous polymers

	Pressure or concentration	Temperature (K)	Capacity (mmol/g)		Reference
			Dry	Humid	
Metal-Organic Frameworks					
MOF-5	9900 ppm	298	0.35	-	19
IRMOF-3	9900 ppm	298	6.18	-	19
MOF-177	9900 ppm	298	2.47	-	19
IRMOF-62	9900 ppm	298	1.35	-	19
MOF-199/HKUST-1	9900 ppm	298	5.12	-	19
	1440 ppm	293	6.6	8.9	16
	1000 ppm	298	6.76	10.12	20
Zn-MOF-74	9900 ppm	298	5.47	-	19
	1440 ppm	293	3.7	2.8	17
Mg-MOF-74	1440 ppm	293	7.6	1.7	17
Co-MOF-74	1440 ppm	293	6.7	4.3	17
Ni-MOF-74	1440 ppm	293	2.3	1.9	17
Cu-MOF-74	2880 ppm	293	3.4	7.6	21
Fe-MIL-100	1000 ppm	298	4.34	2.34	22
DUT-6/MOF-205	1 mbar	298	0.8	-	23
DUT-6-$(OH)_2$	1 mbar	298	4.7	-	23
UiO-66-NH_2	2880 ppm	293	3.3	2.9	24
	1 mbar	298	1.26	-	6
	1440 ppm	293	3.56	3.01	25
	1440 ppm	293	4.04	3.43	26
UiO-66-NH_3Cl	1 mbar	298	2.97	-	6
Fe-MIL-101-SO_3H	1 mbar	298	3.87	--	6
DMOF	1440 ppm	293	0.27	5.56	26
DMOF-NH_2	1440 ppm	293	1.23	1.29	26
ZnMOF	1440 ppm	293	0.25	2.59	26
CuMOF	1440 ppm	293	0.2	0.144	26
Cu-BTB	1440 ppm	293	2.19	5.95	26

Table S3 Continued

	Pressure or concentration	Temperature (K)	Capacity (mmol/g)		Reference
			Dry	Humid	
UiO-66	2880 ppm	293	2.0	-	27
	1440 ppm	293	1.79	2.75	25
ZnBTTB	1440 ppm	293	4.59	20.26	25
DMOF-A	1440 ppm	293	0.48	1.18	25
DMOF-TM2	1440 ppm	293	0.15	4.57	25
UiO-66-NO_2	1440 ppm	293	1.98	1.60	25
UiO-66-OH	2880 ppm	293	5.69	2.77	25
UiO-66-$(OH)_2$	2880 ppm	293	2.29	2.16	25
UiO-66-SO_3H	2880 ppm	293	2.24	1.45	25
UiO-66-$(COOH)_2$	2880 ppm	293	2.83	1.83	25
UiO-66-ox	2880 ppm	293	2.5	-	27
13X Zeolite	1440 ppm	293	2.86	0.62	17
	3 mbar	298	4.94	-	28
		Porous Polymers			
NU-POP-1	1440 ppm	293	5.56	6.17	29
Catechol polymer	1440 ppm	293	0.70	1.31	30
Zn-catecholate polymer	1440 ppm	293	1.36	3.32	30
Cu-catecholate polymer	1440 ppm	293	2.1	4.32	30
Amberlyst 15	1 mbar	298	4.7	-	28
P1-NH_3Cl (BPP-2)	2880 ppm	293	0.7	2.0	*This work*
P1-SO_3H (PPN-6-SO_3H)	2880 ppm	293	3.9	8.1	*This work*
P1-PO_3H_2	2880 ppm	293	5.2	7.2	*This work*
P2-NH_3Cl	2880 ppm	293	1.0	1.5	*This work*
P2-SO_3H	2880 ppm	293	4.0	4.3	*This work*
P2-CO_2H (BPP-7)	2880 ppm	293	6.7	7.4	*This work*

Table S4 NH_3 uptake capacities of metal-organic frameworks and porous polymers at 1 bar

	**Pressure**	**Temperature (K)**	**Capacity (mmol/g)**		**Reference**
			**Dry**	**Humid**	
COF-10	1 bar	298	15	-	31
13X zeolite	1 bar	298	9	-	31
Amberlyst 15	1 bar	298	11	-	31
MCM-41	1 bar	298	7.9	-	31
DUT-6/MOF-205	1 bar	298	12	-	23
DUT-6-$(OH)_2$	1 bar	298	16.4	-	23
UiO-66-NH_2	1 bar	298	10.6	-	6
UiO-66-NH_3Cl	1 bar	298	12.1	-	6
Fe-MIL-101-SO_3H	1 bar	298	17.8	-	6
Prussian blue	1 bar	298	12.5	-	32[a]
$Co[Co(CN)_6]_{0.60}$	1 bar	298	21.9	-	32[a]
$Cu[Fe(CN)_6]_{0.50}$	1 bar	298	20.2	-	32[a]
Mn_2Cl_2(BTDD)	1 bar	298	15.47	-	33[a]
Co_2Cl_2(BTDD)	1 bar	298	12.00	-	33[a]
Ni_2Cl_2(BTDD)	1 bar	298	12.02	-	33[a]
P1-NH_3Cl (BPP-2)	1 bar	298	11.2	-	6
P1-SO_3H (PPN-6-SO_3H)	1 bar	298	12.1	-	6
P1-PO_3H_2	1 bar	298	18.7	-	*This work*
P2-NH_3Cl	1 bar	298	16.3	-	*This work*
P2-SO_3H	1 bar	298	13.1	-	*This work*
P2-CO_2H (BPP-7)	1 bar	298	16.1	-	6

[a] These reports have recently appeared in the literature during the preparation of this manuscript.

9. References

1 W. G. Lu, D. Q. Yuan, D. Zhao, C. I. Schilling, O. Plietzsch, T. Muller, S. Brase, J. Guenther, J. Blumel, R. Krishna, Z. Li and H. C. Zhou, *Chem. Mater.*, 2010, **22**, 5964.

2 A. M. Fracaroli, H. Furukawa, M. Suzuki, M. Dodd, S. Okajima, F. Gandara, J. A. Reimer and O. M. Yaghi, *J. Am. Chem. Soc.*, 2014, **136**, 8863.

3 T. Nakamura, K. Yonesu, Y. Mizuno, C. Suzuki, Y. Sakata, Y. Takuwa, F. Nara and S. Satoh, *Bioorg. Med. Chem.*, 2007, **15**, 3548.

4 T. Ben, H. Ren, S. Q. Ma, D. P. Cao, J. H. Lan, X. F. Jing, W. C. Wang, J. Xu, F. Deng, J. M. Simmons, S. L. Qiu and G. S. Zhu, *Angew. Chem. Int. Ed.*, 2009, **48**, 9457.

5 W. G. Lu, J. P. Sculley, D. Q. Yuan, R. Krishna, Z. W. Wei and H. C. Zhou, *Angew. Chem. Int. Ed.*, 2012, **51**, 7480.

6 J. F. Van Humbeck, T. M. McDonald, X. F. Jing, B. M. Wiers, G. S. Zhu and J. R. Long, *J. Am. Chem. Soc.*, 2014, **136**, 2432.

7 W. G. Lu, D. Q. Yuan, J. L. Sculley, D. Zhao, R. Krishna and H. C. Zhou, *J. Am. Chem. Soc.*, 2011, **133**, 18126.

8 W. Qu, X. Q. Zhu, J. H. Chen, L. Niu, D. H. Liang, X. H. Fan, Z. H. Shen and Q. F. Zhou, *Macromolecules*, 2014, **47**, 2727.

9 O. Plietzsch, C. I. Schilling, M. Tolev, M. Nieger, C. Richert, T. Muller and S. Brase, *Org. Biomol. Chem.*, 2009, **7**, 4734.

10 E. Stockel, X. F. Wu, A. Trewin, C. D. Wood, R. Clowes, N. L. Campbell, J. T. A. Jones, Y. Z. Khimyak, D. J. Adams and A. I. Cooper, *Chem. Commun.*, 2009, 212.

11 R. Dawson, A. Laybourn, R. Clowes, Y. Z. Khimyak, D. J. Adams and A. I. Cooper, *Macromolecules*, 2009, **42**, 8809.

12 Y. Yu, S. L. Jiang and F. Sun, *Ind. Eng. Chem. Res.*, 2014, **53**, 16135.

13 M. Porz, D. Maker, K. Brodner and U. H. F. Bunz, *Macromol. Rapid Commun.*, 2013, **34**, 873.

14 C. Behloul, D. Guijarro and M. Yus, *Synthesis*, 2006, 309.

15 H. P. Ma, H. Ren, X. Q. Zou, S. Meng, F. X. Sun and G. S. Zhu, *Polym. Chem.*, 2014, **5**, 144.

16 G. W. Peterson, G. W. Wagner, A. Balboa, J. Mahle, T. Sewell and C. J. Karwacki, *J. Phys. Chem. C*, 2009, **113**, 13906.

17 T. G. Glover, G. W. Peterson, B. J. Schindler, D. Britt and O. Yaghi, *Chem. Eng. Sci.*, 2011, **66**, 163.

18 J. B. DeCoste and G. W. Peterson, *J. Vis. Exp.*, 2013, e51175.

19 D. Britt, D. Tranchemontagne and O. M. Yaghi, *Proc. Natl. Acad. Sci. U.S.A.*, 2008, **105**, 11623.

20 C. Petit, B. Mendoza and T. J. Bandosz, *Langmuir*, 2010, **26**, 15302.

21 M. J. Katz, A. J. Howarth, P. Z. Moghadam, J. B. DeCoste, R. Q. Snurr, J. T. Hupp and O. K. Farha, *Dalton Trans.*, 2016, **45**, 4150.

22 C. Petit and T. J. Bandosz, *Adv. Funct. Mater.*, 2011, **21**, 2108.

23 I. Spanopoulos, P. Xydias, C. D. Malliakas and P. N. Trikalitis, *Inorg. Chem.*, 2013, **52**, 855.

24 G. W. Peterson, J. B. DeCoste, F. Fatollahi-Fard and D. K. Britt, *Ind. Eng. Chem. Res.*, 2014, **53**, 701.

25 H. Jasuja, G. W. Peterson, J. B. Decoste, M. A. Browe and K. S. Walton, *Chem. Eng. Sci.*, 2015, **124**, 118.

26 B. Mu, PhD thesis, Georgia Institute of Technology, 2011.

27 J. B. DeCoste, T. J. Demasky, M. J. Katz, O. K. Farha and J. T. Hupp, *New J. Chem.*, 2015, **39**, 2396.

28 J. Helminen, J. Helenius, E. Paatero and I. Turunen, *J. Chem. Eng. Data*, 2001, **46**, 391.

29 G. W. Peterson, O. K. Farha, B. Schindler, P. Jones, J. Mahle and J. T. Hupp, *J. Porous Mater.*, 2012, **19**, 261.

30 M. H. Weston, G. W. Peterson, M. A. Browe, P. Jones, O. K. Farha, J. T. Hupp and S. T. Nguyen, *Chem. Commun.*, 2013, **49**, 2995.

31 C. J. Doonan, D. J. Tranchemontagne, T. G. Glover, J. R. Hunt and O. M. Yaghi, *Nat. Chem.*, 2010, **2**, 235.

32 A. Takahashi, H. Tanaka, D. Parajuli, T. Nakamura, K. Minami, Y. Sugiyama, Y. Hakuta, S. Ohkoshi and T. Kawamoto, *J. Am. Chem. Soc.*, 2016, **138**, 6376.

33 A. J. Rieth, Y. Tulchinsky and M. Dincă, *J. Am. Chem. Soc.*, 2016, **138**, 9401.
